# Macrophages derived exosomes deliver miR-223 to epithelial ovarian cancer cells to elicit a chemoresistant phenotype

**DOI:** 10.1186/s13046-019-1095-1

**Published:** 2019-02-15

**Authors:** Xiaolan Zhu, Huiling Shen, Xinming Yin, Meiling Yang, Hong Wei, Qi Chen, Fan Feng, Yueqin Liu, Wenlin Xu, Yuefeng Li

**Affiliations:** 1 Department of Gynecologic Oncology and Central Laboratory, Fourth Affiliated Hospital of Jiangsu University, 20 Zhengdong Road, Zhenjiang, Jiangsu 212001 People’s Republic of China; 2 Reproductive Sciences Institute, Jiangsu University, Zhenjiang, 212001 Jiangsu China; 3 Department of Oncology, Affiliated People Hospital of Jiangsu University, Zhenjiang, 212001 Jiangsu China; 4 Department of Radiology and Pathology, Affiliated Hospital of Jiangsu University, 438 Jiefang Road, Zhenjiang, Jiangsu 212001 People’s Republic of China

**Keywords:** Exosome, miR-223, Chemoresistance, Macrophages, EOC

## Abstract

**Background:**

How exosomal microRNAs (miRNAs) derived from macrophages contribute to the development of drug resistance in the context of the hypoxic tumor microenvironment in epithelial ovarian cancer (EOC) remains poorly understood.

**Methods:**

The miRNA levels were detected by qRT-PCR. Protein levels of HIF-1α, CD163 and PTEN-PI3K/AKT pathway were assessed by Western blot (WB) and Immunohistochemistry (IHC). Exosomes were isolated, and then confirmed by Transmission electron microscopy (TEM), Nanoparticle Tracking Analysis (NTA) and WB. Internalization of macrophages-secreted exosomes in EOC cells was detected by Confocal microscope. Subsequently, Dual-luciferase reporter assay verified PTEN was the target of miR-223. Gain- and loss-of-function experiments, rescue experiments, and SKOV3 xenograft models were performed to uncover the underlying mechanisms of miR-223 and PTEN-PI3K/AKT pathway, as well as the exosomal miR-223 in inducing multidrug resistance in vitro and in vivo.

**Results:**

Here, we showed hypoxic EOC cells triggered macrophages recruitment and induced macrophages into a tumor-associated macrophage (TAM)-like phenotype; exosomes derived from hypoxic macrophages enhanced the malignant phenotype of EOC cells, miR-223 was enriched in exosomes released from macrophages under hypoxia, which could be transferred to the co-cultivated EOC cells, accompanied by enhanced drug resistant of EOC cells. Besides, results from a functional assay revealed that exosomal miR-223 derived from macrophages promoted the drug resistance of EOC cells via the PTEN-PI3K/AKT pathway both in vivo and in vitro. Furthermore, patients with high HIF-1a expression had statistically higher CD163+ cell infiltration and intertumoral levels of miR-223. Finally, circulating exosomal miR-223 levels were closely related to the recurrence of EOC.

**Conclusions:**

These data indicate a unique role of exosomal miR-223 in the cross-talk between macrophages and EOC cells in chemotherapy resistance, through a novel exosomal miR-223/PTEN-PI3K/AKT signaling pathway.

**Electronic supplementary material:**

The online version of this article (10.1186/s13046-019-1095-1) contains supplementary material, which is available to authorized users.

## Introduction

Despite all current standard treatments, the prognosis of patients with high-risk ovarian cancer is still poor, the main reason for failure is the development of chemoresistance [1]. Cancer is not merely a cell-intrinsic genetic disease, but also the result of complex cell-extrinsic interactions with host components, including surrounding stromal cells [2]. Macrophages are one of the major populations of tumor infiltrating immune cells and represent the most abundant host cell population within tumor stroma [3]. The resident macrophages of tumor stroma exhibiting protumor functions are often termed as tumor associated macrophages (TAMs), which display M2 like phonotype [4]. The positive correlation and ongoing dynamic interactions between tumor cells (including ovarian cancer) and TAMs has been revealed by clinical studies [5–7]. Tumor cell oxygen deficiency represents a key micro-environmental stressor governing multiple phenomenon associated with tumor progression, while macrophages are known to preferentially concentrate in hypoxic areas of tumors, and hypoxic tumor milieu has been proposed to be the most probable cause of phenotype switching [8, 9]. Consistent with this, the hypoxic area of human ovarian carcinomas are known to have large congregation of M2 like TAMs [6]. Although the evidences in support of hypoxic microenvironment being instrumental in macrophage recruitment and polarization are gradually mounting, the exact way and content of interaction between macrophages and EOC cells during hypoxia is poorly understood and warrants an in-depth investigation.

An important mode of communication between carcinoma cells and immune cells may be the membrane-bound packets called exosomes, whose size ranges from 20 to 100 nm and particularly enriched in various tumor microenvironments [10, 11]. Hypoxia was shown to enhance exosomes release [12], and to induce increased shedding of pro-angiogenic MVs (micro-vesicles) [13]. Tumor-derived exosomes are known to be involved in chemoresistance in many cancers, including ovarian cancer [14, 15]. These released molecules can then circulate throughout the body and are protected from degradation, mainly because of their powerful regulation of gene expression and comparably stable characteristics. Among the cargos carried by exosomes are small molecules of RNA known as microRNAs, which post-transcriptionally control the translation and stability of mRNAs [10]. Shurtleff et al. confirmed that one exosome-specific microRNA, miR-223, was selectively packed into exosomes and was much more abundant in the exosomes than the cells [16]. While broadly expressed in myeloid cells, miR-223, highly expressed in the human macrophage cell line THP-1 and primary macrophages [5, 16, 17]. In fact, this microRNA has emerged as a putative predictor of tumor invasiveness, metastasis and recurrence in many tumor types [18, 19].

As a critical regulator of the PI3K/AKT signal transduction pathway, PTEN controls multiple cellular responses, including cell survival [20]. Activation of PI3K by extracellular stimuli generates phosphatidylinositol triphosphate (PIP3) in the plasma membrane. PIP3 recruits the AKT kinase to the membrane, where it is phosphorylated and activated by phosphatidylinositol-dependent kinase-1 [21]. Upon its activation, AKT triggers the phosphorylation of numerous protein targets, and is consequently associated with a poor prognosis of ovarian cancer and initiates downstream targets that modulate multiple cellular processes including cell proliferation [20, 22].

Despite a growing number of studies demonstrating that TAM infiltration plays a role in tumor recurrence, the biological activities of the TAM-released exosomes communicated with EOC cells under ovarian cancer hypoxia microenvironment remains less well studied and needs detailed investigation. In particular, how exosomal miRNAs released within the tumor microenvironment (TME) affect resistance to chemotherapy is still unknown. We hypothesize that TAMs affect EOC resistance to chemotherapy through the exchange of exosomal miRNAs, so this study aims to study whether exosomal miR-223 is involved, and through which molecular mechanisms it elicits this function.

## Materials and methods

### Cell culture and treatment

The human epithelial ovarian cancer cell line A2780 was obtained from NANJING KEYGEN BIOTECH CO., LTD, China. SKOV3 cells, human breast cancer cell line (MCF-7, MBA-MD-231), human cervical cancer cell line (Hela), Human leukemia monocyte THP1 cell line were obtained from the Shanghai Institute of Cell Biology at the China Academy of Sciences. The human epithelial ovarian cancer cell lines (A2780 and SKOV3), MCF-7 and THP1 cells were authenticated by Short Tandem Repeat assay (STR). All cells used were passaged less than 3 months after resuscitation. The culture conditions and treatment are described in the Additional file 1: Supplementary methods.

### Tissue samples

With the approval of the Jiangsu University Ethics Committee, serous ovarian cancer samples from patients with FIGO stage IIIC or IV (*n* = 62) were collected at Zhenjiang Maternal and Child Health Hospital (The Fourth Affiliated Hospital of Jiangsu University) and The Affiliated Hospital of Jiangsu University. All patients were treated with the standard care of platinum-based therapy after surgery, and informed consent was obtained from all patients. Sera of 12 patients who received chemotherapy after first surgery and relapse within 6 months were available at the time of surgery and after recurrence. PFS was calculated from the time of surgery to the time of progression or recurrence. Platinum resistance or platinum sensitivity was defined by relapse or progression within 6 months or 6 months after the last platinum-based chemotherapy, respectively. Clinical and pathological features are described in Additional file 2: Table S1.

### Exosome collection, characterization, quantification, labeling and tracking

The details were described in in the Additional file 1: Supplementary methods.

### Transfection

The details are described in the Additional file 1: Supplementary methods.

### Reverse transcription quantitative real-time PCR

The details are described in the Additional file 1: Supplementary methods.

### Western blot analysis

The details are described in the Additional file 1: Supplementary methods.

### Luciferase reporter assays

Luciferase assays were performed using the Dual-Luciferase Reporter assay system (Promega, Madison, WI, USA) as described previously [23]. The detailed procedures and materials are described in the Additional file 1: Supplementary methods.

### Flow cytometric analysis

The details are described in the Additional file 1: Supplementary methods.

### Evaluation of apoptosis and assessing chemosensitivity to cDDP

The details are described in the Additional file 1: Supplementary methods.

### Cell migration assays

The details are described in the Additional file 1: Supplementary methods.

### ELISA assay

The cell culture medium of macrophages, which were stimulated with EOC cells-conditioned media, was collected and measured using IL-10 and IL12p70 ELISA kits (Ray Biotech, USA).

### Animal assay

The female athymic nude mice aged 4 weeks were purchased from School of Medicine, Shanghai Jiao Tong University (Shanghai, China) and maintained under SPF conditions. All the animal experiments were manipulated according to guidelines approved by the Jiangsu University Medical Experimental Animal Care Commission. Mice were irradiated with 2 Gy total body irradiation to achieve a more complete immunosuppression, avoid murine macrophage infiltration, and allow better xenograft growth, as previously described [24]. SKOV3 (1.0 × 10^6^) were injected intraperitoneally into the mice which were then randomly divided into five groups (six animals per condition). TAMs were transfected with miR-223 agomir or antagomir under normoxic and hypoxic conditions respectively. When mice had palpable tumors (about a week after injection), cDDP (5 mg/kg) and TAMs exosomes (10 μg) or PBS were then injected into the center of the xenograft tumors twice per week for 3 consecutive weeks. On day 28, mice were sacrificed and tumors were harvested.

### Immunohistochemistry and scoring

The details are described in the Additional file 1: Supplementary methods.

### Statistical analyses

The data were presented as mean ± SD from at least three independent experiments. The comparisons of means among groups were analyzed by oneway ANOVA, and the Dunn Multiple Comparison Test was further used to determine significant differences between groups at the significance level of *P* < 0.05 (*P < 0.05 and **P < 0.01). Survival curves were examined by Kaplan-Meier curves with the log-rank test and Cox proportional hazard analysis. Spearman’s non-parametric correlation test was performed to test the correlation between the expression levels of miR-223 and PTEN by GraphPad Prism 5 (GraphPad Software, Inc., La Jolla, CA, USA).

## Results

### Hypoxic EOC cells trigger macrophages recruitment and their M2 polarization

**Fig. 1:**
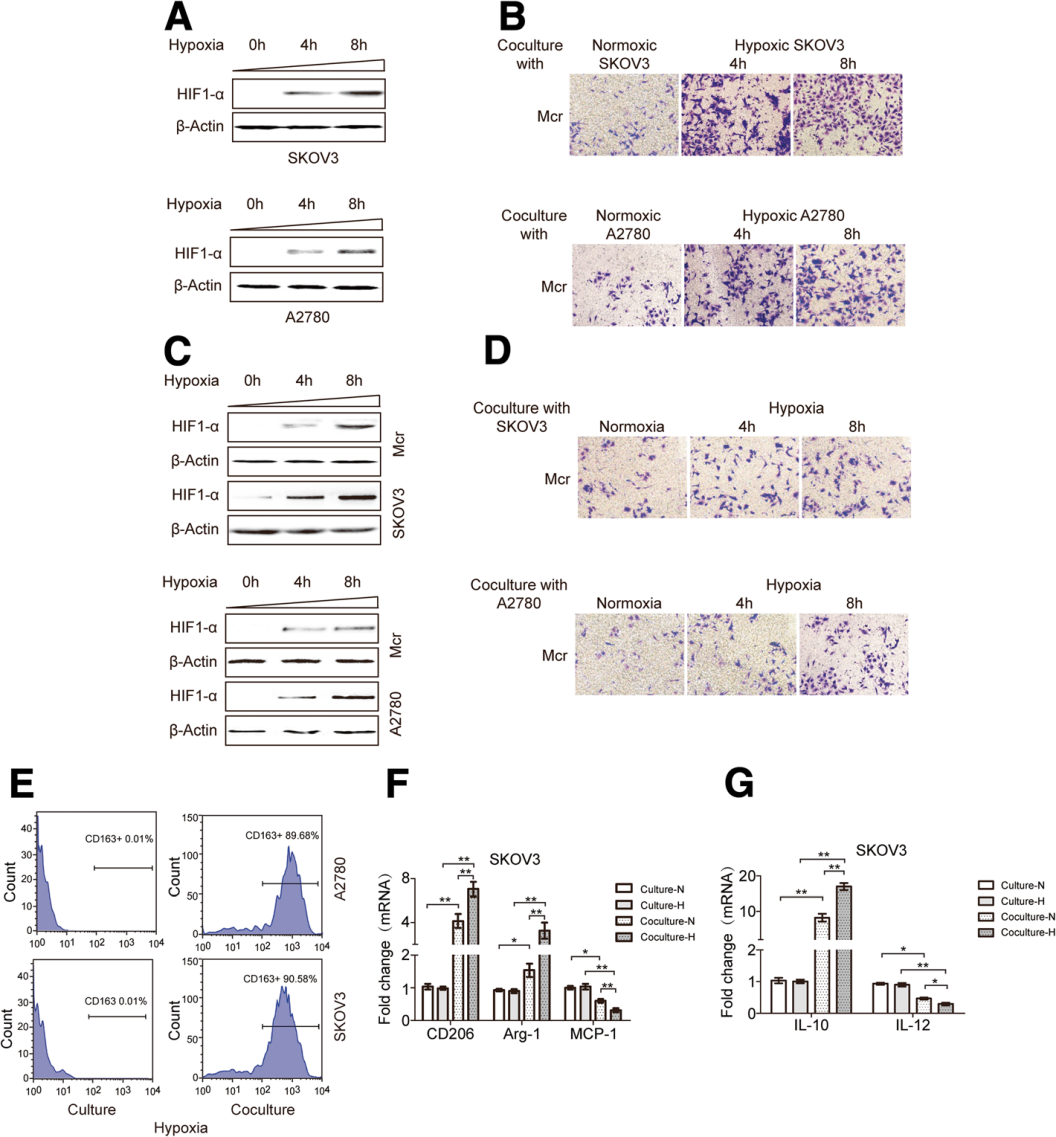
Hypoxic EOC cells induced macrophages into a TAM-like phenotype. **a** Changes of HIF-1α expression in EOC cells in exposure to hypoxia for 4 or 8 h. **b** After cocultured with normoxic or hypoxic EOC cells (exposure to hypoxia for 4 or 8 h) for 24 h, migration ability of macrophages was detected (× 200 magnification). **c** HIF-1α expression in EOC cells and macrophages after exposure to hypoxia together for 4 or 8 h. **d** After exposure to hypoxia for 4 or 8 h, macrophages were cocultured with normoxic or hypoxic EOC cells for an additional 24 h, then, migration ability of macrophages was detected. **e** Flow cytometry expression of CD163 in macrophages alone (control, left panels) or cocultured with EOC cells (coculture, right panels) for 48 h. **f** The related fold change of CD206,Arg-1 and MCP-1 mRNA in macrophages alone or cocultured with normoxic or hypoxic SKOV3 cells. **g** IL-10 and IL-12 expression levels in macrophages were evaluated by ELISA. *P < 0.05, **P < 0.01

Human THP-1 monocytes were differentiated into macrophages by a 24 h incubation in the presence of 100 ng/ml phorbol 12-myristate 13-acetate (PMA). After differentiation, round and floating THP-1 cells became adherent flattened cells (Data not shown). To test whether EOC cells in response to hypoxic stress trigger macrophages recruitment, we evaluated the transmigration of macrophages towards EOC cells that were exposed to hypoxic conditions for 4 and 8 h previously. Then, HIF-1α was detected to test whether the experimental conditions could successfully elicit hypoxic stress in EOC cells. As showed, HIF-1α expression exhibited a time dependent increase in response to hypoxia (Fig. 1a). The non-contact cocultures of macrophages (upper) and EOC cells (lower) were maintained for 24 h to observe the migration of macrophages towards hypoxic and normoxic EOC cells. As showed in (Fig. 1b), the number of transmigrated macrophages were positively correlated with the duration of EOC cells exposure to hypoxia. However, when EOC cells and macrophages were exposed to hypoxia together for 4 and 8 h, the macrophages migrated at a much smaller scale after an additional 24 h coculture (Fig. 1c and d). That was consistent with the previous report revealing impaired migratory potential of macrophages in hypoxic conditions [25].

TAMs exhibit an M2 polarized phenotype. Next, we asked whether EOC cells induce M1- or M2-polarization of unpolarized macrophages hypoxia. By performing cytofluorimetry for the TAM marker CD163 in macrophages, we observed an increased percentage of CD163+ cells when unpolarized macrophages were cocultured with normoxic and hypoxic EOC cells (Fig. 1e and Additional file 3: Figure. S1A). The expression of M1 and M2 typical markers was detected in macrophages, and increased trends of CD206 and Arg-1 whereas reduced MCP-1 was found in macrophages cocultured with both normoxic and hypoxic EOC cells (Fig. 1f and Additional file 3: Figure S1B). Consistent with the changes in surface markers, macrophages cocultured with EOC cells produced a higher level of IL-10 and a lower level of IL-12 (Fig. 1g and Additional file 3: Figure S1C). However, these phenomena were more obvious when macrophages cells were cocultured with hypoxic EOC cells. These data indicates macrophages cocultured with EOC cells had an M2-skewed phenotype.

### Hypoxia stimulation enhances internalization of macrophages-secreted exosomes in EOC cells

**Fig. 2:**
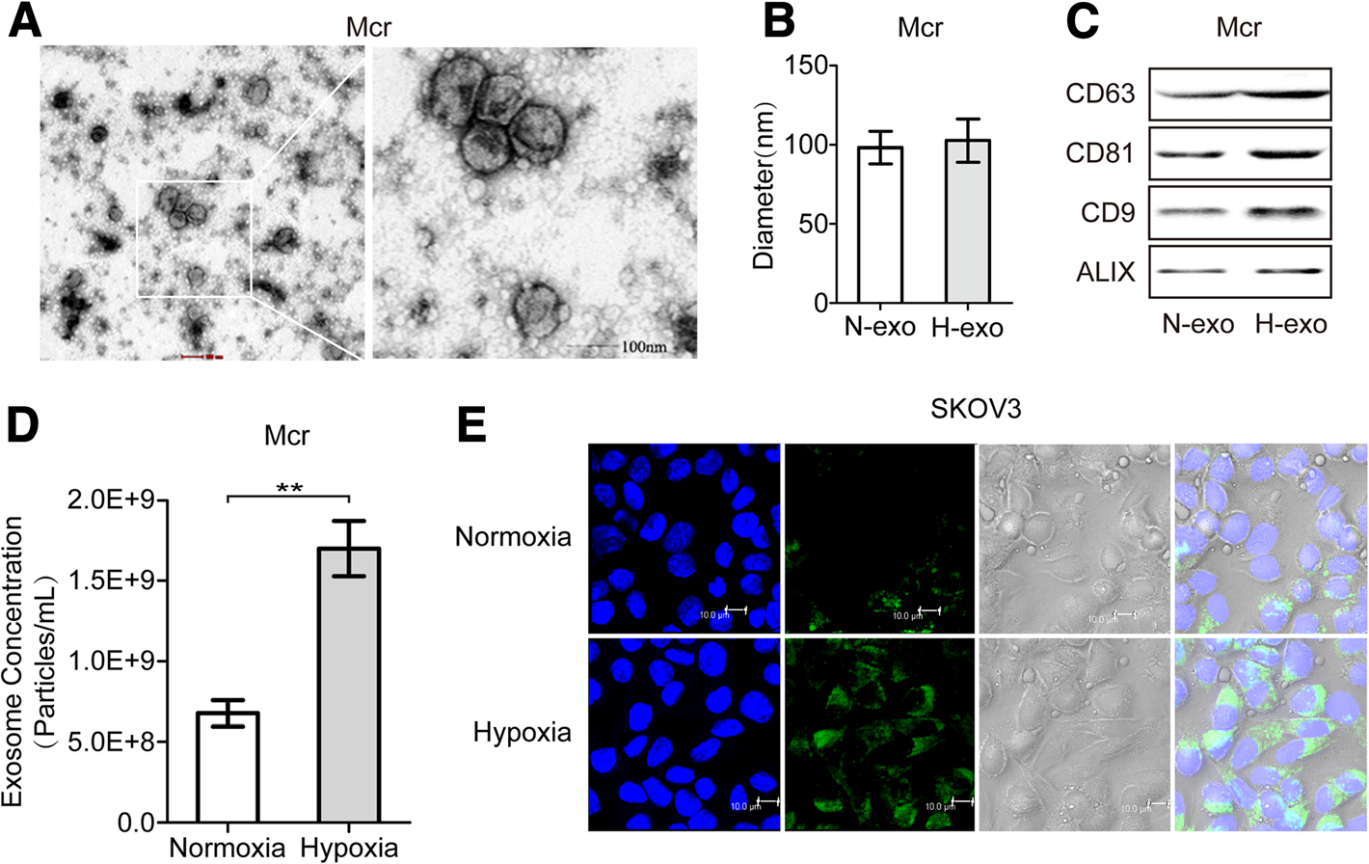
Hypoxia stimulation enhances internalization of macrophages-secreted exosomes by EOC cells. **a** Representative electron micrograph of exosomes isolated from macrophages-conditioned medium revealing the typical morphology and size (50–200 nm). Scale bar, 100 nm. **b** The diameter of exosomes (exo) derived from the conditioned medium of normoxic (N-exo) or hypoxic (H-exo) macrophages. **c** Western blot analysis showing the presence of CD63, CD81, CD9 and ALIX in the exosomes. **d** The concentration of exosomes was detected. **e** Representative confocal microscopy images showing the internalization of PKH67-labeled exosomes (green) derived from macrophages by SKOV3 cells under normoxia or hypoxia. Scale bar, 10 μm. *P < 0.05, **P < 0.01

Because there is a correlation between the amount of exosomes released and oncogenesis, and hypoxia was shown to enhance exosomes release by cancer cells and to induce increased shedding of pro-angiogenic MVs from glioma cells [26, 27]. We first examined the secretion of TAM exosomes under hypoxia: macrophages were polarized to TAMs by IL4, the purified exosomes from TAM were examined by SEM, which revealed the presence of exosomes within the expected size range of exosomes (50–150 nm) and bilayer cup shaped morphology (Fig. 2a); then we evaluated the role of hypoxia on TAMs exosomes release: equivalent numbers of TAMs were exposed to normoxia or hypoxia for 24 h. Nanoparticle-tracking analysis (NTA) on the isolated samples derived from hypoxic or normoxic conditions indicated that most of the particles had a similar size of 50-150 nm in diameter with a peak at around 100 nm (Fig. 2b). In addition, the production of exosomes was quantified by Western blot and NTA analyses, all exosomal markers (CD63, CD81, CD9 and ALIX) were higher and exosomes secretion was significantly increased in exosomes’ fractions prepared from hypoxic TAMs being compared with normoxic cells (Fig. 2c and d), suggesting a greater number of exosomes were present in hypoxic TAMs. Further, to determine whether exosomes could be taken up by the recipient EOC cells, SKOV3 cells were incubated with PKH67 labeled exosomes that were isolated from TAMs, and then the internalization of TAMs exosomes in EOC cells was monitored by confocal microscopy. With the extension of coculture time, uptake of TAMs derived exosomes by EOC cells increased significantly under hypoxic conditions (Fig. 2e and Additional file 4: Figure S2A and B). To rule out contamination of unbound PKH67 dye and non-specific transfer to SKOV3 cells, a PKH labeling control was utilized during the PKH staining procedure by including a tube without exosomes, to which PKH dye was added. No increased fluorescence was observed following incubation with the PKH labeling control (Data not shown), confirming the specificity of the detected exosome uptake.

### TAMs exosomes confer drug resistance of recipient EOC cells

**Fig. 3:**
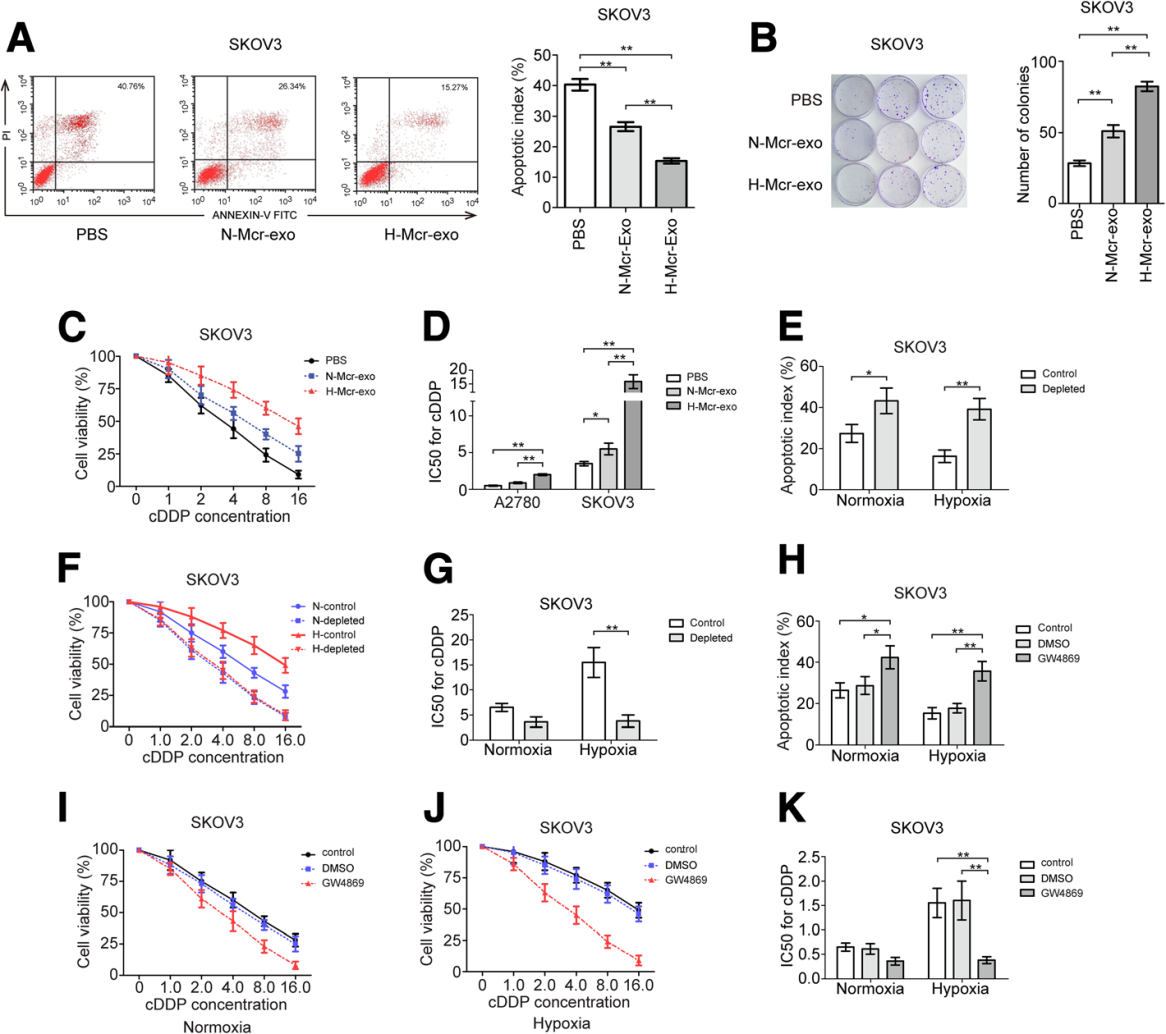
TAMs derived exosomes confer drug resistance of recipient EOC cells. SKOV3 cells were treated with PBS, exosomes derived from normoxic (N-Mcr-exo) or hypoxic (H-Mcr-exo) TAMs for 24 h, cell apoptosis and related quantitative analysis of apoptosis (**a**), the number of cell colonies (**b**), cell viability (**c**), IC50 for cDDP (**d**) was analyzed in SKOV3 cells respectively. SKOV3 cells were cultured in TAMs-conditioned and exosome-depleted TAMs-conditioned media, cell apoptosis (**e**), cell viability (**f**), IC50 for cDDP (**g**) was measured in SKOV3 cells. SKOV3 cells were treated with DMSO or GW4869, cell apoptosis (**h**), cell viability (**i**, **j**), IC50 for cDDP (**k**) was detected. *P < 0.05, **P < 0.01

Once secreted, exosomes deliver biologic information by internalization by neighboring or distant cells. To determine the effects of exosomes derived from TAMs on the malignant phenotype of EOC cells, the EOC cells were treated with PBS, and exosomes derived from normoxic or hypoxic TAMs. As shown, the exosomes of TAMs reduced apoptosis rate, increased the number of cell colonies and cell viability, and enhanced drug resistance of EOC cells. Importantly, hypoxic TAMs derived exosomes showed greater ability to induce cell proliferation and inhibit apoptosis of EOC cells compared with exosomes secreted from normoxic TAMs (Fig. 3a-d and Additional file 5: Figure S3A and B). Next, we assessed whether physically removing exosomes from TAMs-conditioned media would affect their ability to increase chemoresistance, and we found that depletion of exosomes by ultracentrifugation from TAMs-conditioned media significantly reduced cell viability and decreased drug resistance in recipient EOC cells receiving the TAMs-conditioned media (Fig. 3e-g and Additional file 5: Figure S3C-E). In addition, we utilized GW4869 to inhibit neutral sphingomyelinase and prevent exosomes release in TAMs that were cocultured with EOC cells. As expected, increased apoptosis rate, reduced cell viability and decreased drug resistance of EOC cells were observed following GW4869 treatment (Fig. 3h-k and Additional file 5: Figure S3F-I). These findings indicate that TAMs enhance the malignant phenotype of EOC cells by transmitting exosomes.

### Identification of the EOC-TAMs exosomal miR-223 crosstalk

**Fig. 4:**
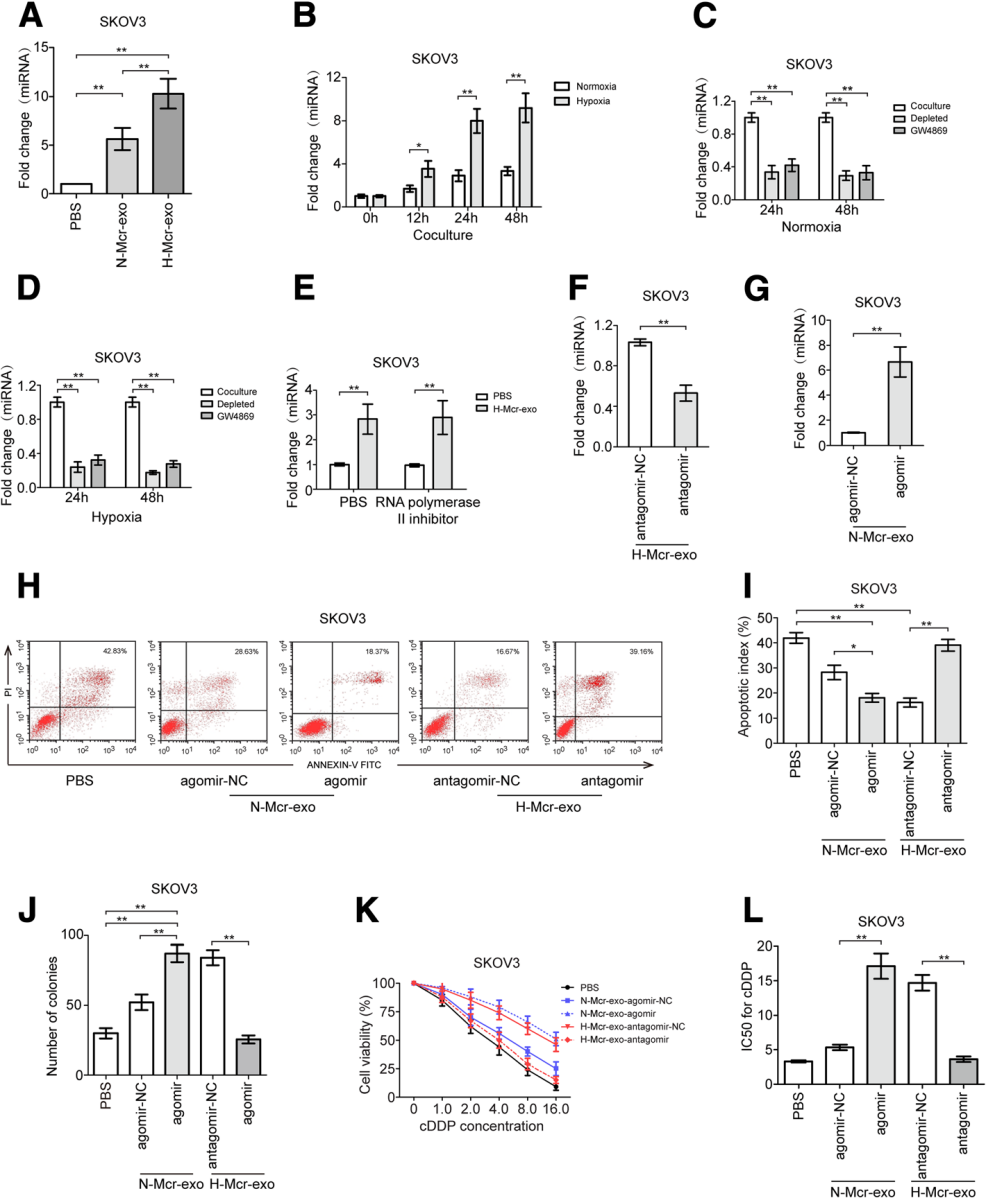
Exosomic miR-223 mediated cross-talk between macrophages and EOC cells. **a** Related miR-223 level in SKOV3 cells treated with PBS, N-Mcr-exo or H-Mcr-exo. **b** Fold change of miR-223 in SKOV3 cells cocultured with macrophages for the indicated time periods. **c**, **d** Fold change of miR-223 in SKOV3 cells cocultured with TAMs-conditioned, exosome-depleted TAMs-conditioned media or TAMs-conditioned media pretreated with GW4869 under normoxia (**c**) and hypoxia (**d**). **e** qRT-PCR for mature miR-223 in SKOV3 cells treated with RNA polymerase II inhibitor or PBS. **f**-**k** SKOV3 cells were treated with exosomes derived from the normoxic or hypoxic macrophages which were transfected with agomir or antagomir respectively, the related miR-223 level (**f**, **g**), cell apoptosis (**h**, **i**), the number of cell colonies (**j**), cell viability (**k**), IC50 for cDDP (**l**) was measured. *P < 0.05, **P < 0.01

The exosomal transfer of miRNAs was regarded as a novel and important mechanism of genetic exchange between cells [28]. By qRT-PCR, we validated that miR-223 was increased in TAMs and exosomes secreted by them under hypoxia (Additional file 6: Figure S4A). miR-223 but not pre-miR-223 levels were statistically significantly increased in EOC cells cocultured with hypoxic TAMs or exosomes derived from hypoxic TAMs (Fig. 4a and b and Additional file 6: Figure S4C-E). Similarly, when EOC cells were cultured with the TAMs pretreated with GW4869 or exosome-depleted TAMs-conditioned media, mature miR-223 levels were significantly reduced (Fig. 4c and d and Additional file 6: Figure S4F and G). In addition, the increase of miR-223 in EOC cells exposed to hypoxic TAMs exosomes was not prevented by an RNA polymerase II inhibitor (Fig. 4e and Additional file 6: Figure S4H). These data reveal that hypoxia stimulation enriches miR-223 in TAMs and the exosomes secreted by them, so as in recipient EOC cells.

HIF-1α was the major transcription factors that was responsive to hypoxia in mammalian cells [29]. Thus, we examined if HIF-1α was involved in the increased exosmic miR-223 production in TAMs during hypoxia. We initially tested the effects of DMOG (pharmacological inducer of HIF-1α) and YC-1 (pharmacological inhibitor of HIF-1α) respectively with or without hypoxia treatment for 24 h. DMOG increased, while YC-1 reduced miR-223 expression both in TAMs and TAMs secreted exosomes (Additional file 6: Figure S4I and J). These data suggest the possibility of the involvement of HIF-1α in the production of miR-223 in TAMs cells and the exosomes they secreted.

To study the function of exosomal miR-223, we inhibited miR-223 by antagomir and overexpressed miR-223 using the agomir in TAMs. The inhibition of miR-223 resulted in a decrease of miR-223 in hypoxic TAMs derived exosomes (Fig. 4f and Additional file 6: Figure S4K), while the overexpression by miR-223 resulted in an increase of miR-223 in normoxic TAMs derived exosomes, as determined by qRT-PCR (Fig. 4g and Additional file 6: Figure S4L). Then, SKOV3 cells were treated with normoxic and hypoxic exosomes derived from the wild-type, agomir and antagomir transfected TAMs respectively. As shown in Fig. 4h-l and Additional file 6: Figure S4M, the upregulation of miR-223 significantly reduced apoptosis rate, increased the number of cell colonies and cell viability, enhanced drug resistance of target SKOV3 cells compared with wild-type exosomes. Consistently, decreased miR-223 caused increased apoptosis rate, reduced cell viability and impaired drug resistance of SKOV3 cells. In summary, hypoxic TAMs derived exosomes containing miR-223 were internalized by EOC cells and promoted drug resistance of EOC cells.

### Exosmic miR-223 inactivate PI3K/AKT pathway through PTEN targeting

**Fig. 5:**
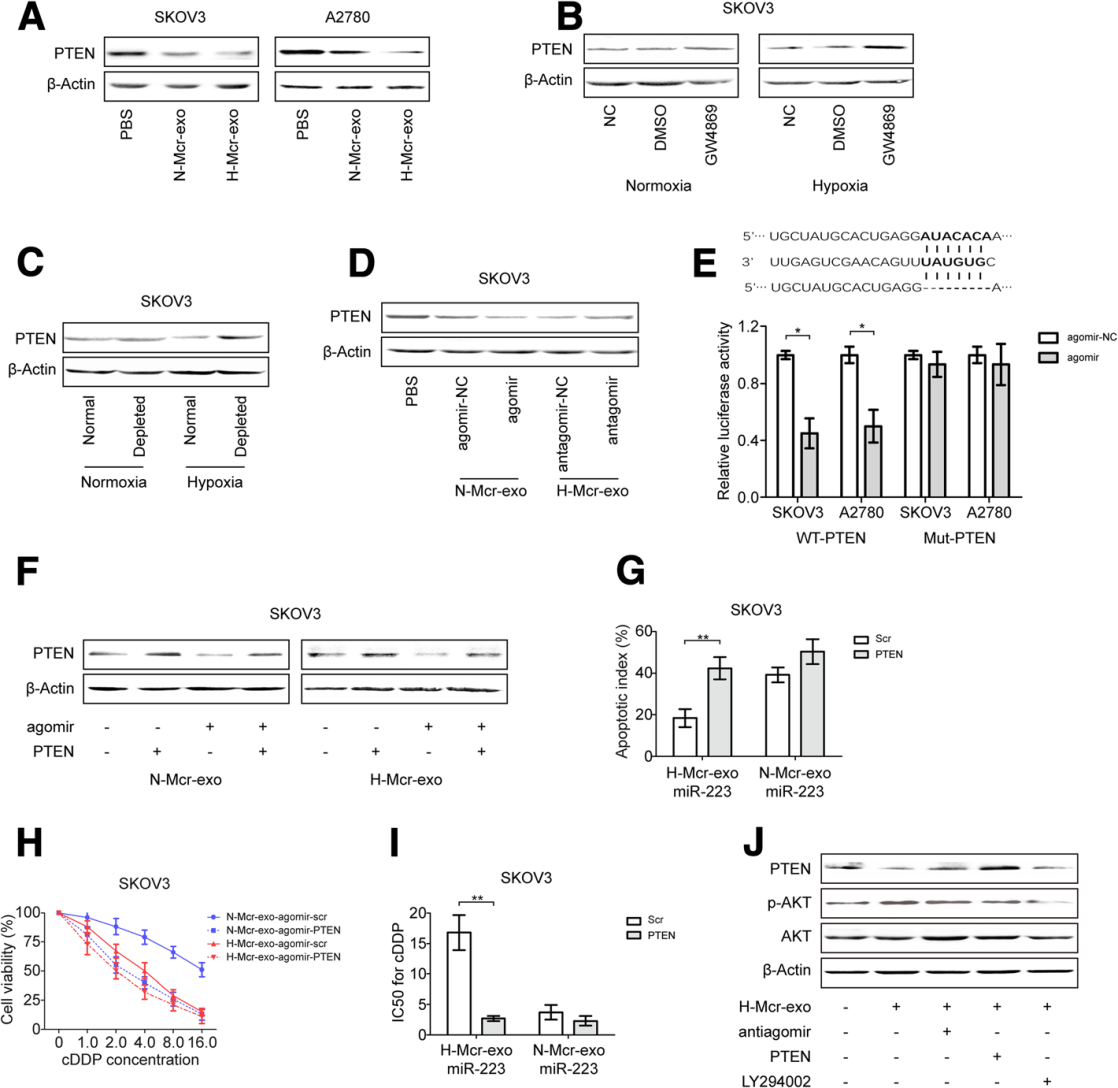
Exosmic miR-223 inactivates PI3K/AKT pathway through PTEN targeting. **a** Detection of PTEN protein level (by immunoblotting) in EOC cells treated with PBS, N-Mcr-exo or H-Mcr-exo. **b** PTEN expression in SKOV3 cells cocultured with TAMs-conditioned media pretreated with DMSO or GW4869 under normoxia and hypoxia. **c** PTEN expression in SKOV3 cells cocultured with TAMs-conditioned, exosome-depleted TAMs-conditioned media under normoxia and hypoxia. **d** PTEN expression in SKOV3 cells treated with exosomes derived from the normoxic or hypoxic macrophages which were transfected with agomir or antagomir respectively. **e** Luciferase reporter assay in EOC cells cotransfected with wild-type (WT) or mutant (Mut) PTEN 3’UTR reporter gene and agomir or agomir-NC. **f**-**i** SKOV3 cells transfected with a plasmid expressing PTEN, then treated with exosomes derived from the normoxic or hypoxic macrophages which were transfected with agomir, PTEN protein level (**f**) cell apoptosis (**g**),cell viability (**h**), IC50 for cDDP (**i**) was measured. **j** PTEN, p-AKT and AKT protein levels in SKOV3 cells treated as indicated. *P < 0.05, **P < 0.01

Next, we validated that PTEN expression in EOC cells was down-regulated by hypoxic TAMs derived exosomes (Fig. 5a). Then, we treated SKOV3-TAMs cocultures with GW4869, or DMSO as a control. Only GW4869 induced significantly upregulation of PTEN in SKOV3 cells (Fig. 5b). Moreover, increased PTEN was detected in EOC cells cocultured with exosome-depleted TAMs-conditioned media compared with TAMs-conditioned media (Fig. 5c). The down-regulation of PTEN by TAMs exosomes was enhanced when normoxic TAMs were pretreated with agomir, and reversed when hypoxic TAMs were pretreated with antagomir (Fig. 5d). Further, we performed a luciferase reporter assay in two different EOC cell lines and observed direct targeting of PTEN by miR-223 (Fig. 5e). We transfected EOC cells with a plasmid expressing miR-223-not-regulated PTEN (no-3’UTR). Consistent with the results from the reporter assay, the transfection inhibited the downregulation of PTEN caused by TAMs exosomes and miR-223 (Fig. 5f), along with increased apoptosis rate, reduced cell viability and decreased drug resistance of target SKOV3 cells (Fig. 5g-i). These data indicates that PTEN is able to reverse the cDDP-resistant phenotype induced by exosomal miR-223 in EOC cells. Finally, we assessed the expression of miR-223 and PTEN in different cancer cell lines cocultured with PBS or hypoxic TAMs derived exosomes. In all cases, we observed upregulation of miR-223 and downregulation of PTEN, suggesting that the exosomal miR-223 targeting of PTEN in cancer cells-TAMs cocultures could also occur in other cancer types (Additional file 7: Figure S5A and B).

To pursue this putative link between miR-223/PTEN and the PI3K/AKT pathway in EOC cells, we examined the levels of AKT phosphorylation in EOC cells. The abundance of p-AKT (phosphorylated on Ser473) were up-regulated and inversely correlated with PTEN expression in hypoxic TAMs exosomes treated EOC cells (Fig. 5j). In contrast, miR-223 inhibition, PTEN-expressing and AKT signaling pathway blocking EOC samples (all pretreated with H-Mcr-exo) had low levels of AKT phosphorylation, indicating the inactivation of the PI3K/AKT signaling pathway. To test whether the activation of the PI3K/AKT signaling pathway was involved in EOC cells resistance to chemotherapy drugs, we examined the effect of the presence or absence of LY294002 on the sensitivity of SKOV3 cells. As showed, LY294002 was sufficient to overcome H-Mcr-exo-induced cDDP resistance in SKOV3 cells (Additional file 7: Figure S5C). The data suggest that H-Mcr-exo-mediated chemoresistance phenotype observed in EOC cells may be associated with the downregulation of PTEN, which activated the downstream signal transduction pathways including the PI3K/AKT pathway.

### Effects of TAMs-derived exosomal miR-223 on EOC drug resistance in vivo

**Fig. 6:**
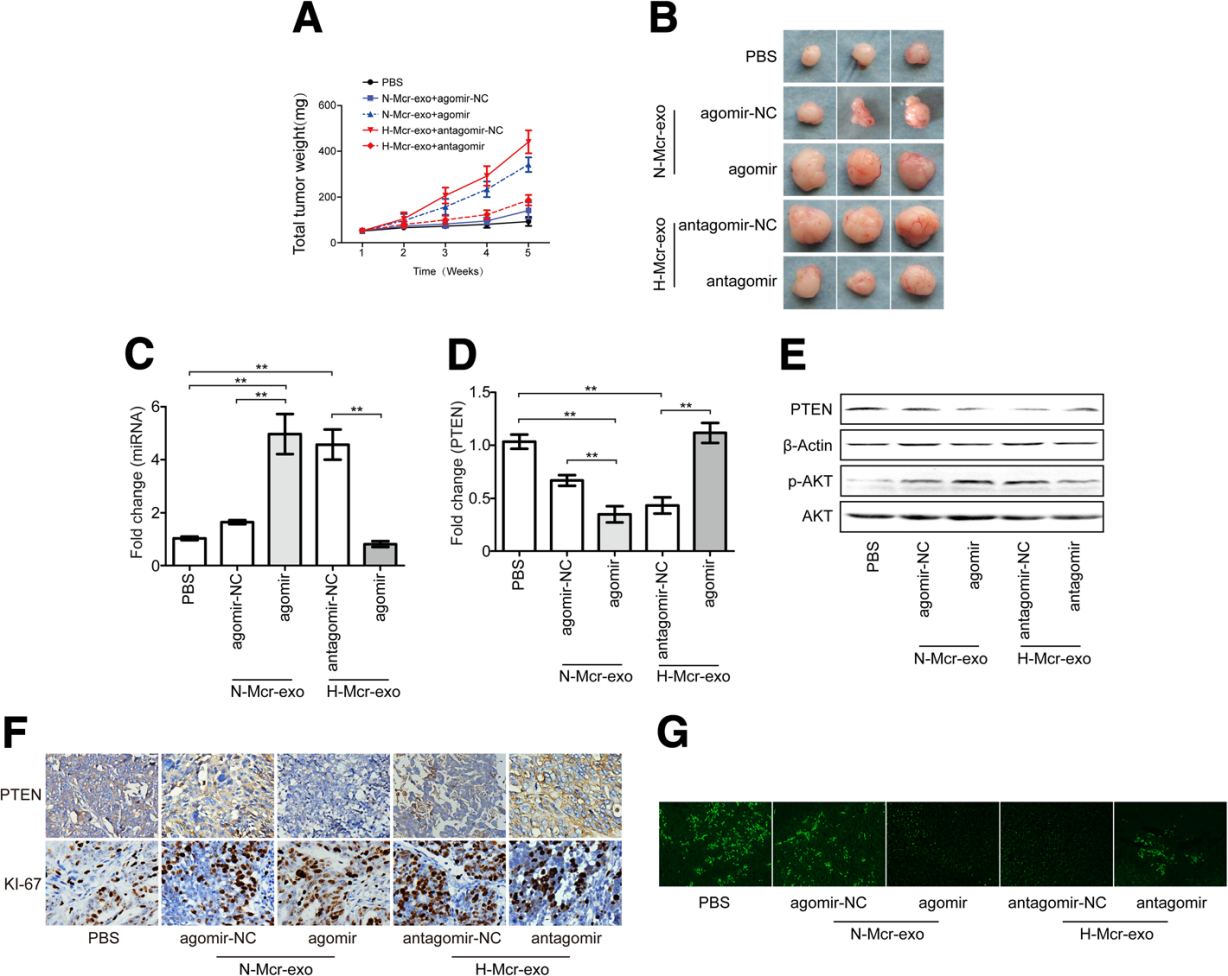
Effects of exosomic miR-223 in cisplatin resistance in vivo. Exosomes derived from the normoxic or hypoxic macrophages which were transfected with agomir or antagomir respectively (N-Mcr-exo + agomir-NC, N-Mcr-exo + agomir, H-Mcr-exo + antagomir-NC, H-Mcr-exo + antagomir). SKOV3 cells were subcutaneously injected into BALB/c nude mice, we allowed potential tumors to grow for a week, then, cDDP (5 mg/kg) and exosomes (10 μg) or PBS were injected into the center of the xenograft tumors twice per week for 3 consecutive weeks (*n* = 6). **a** Growth curves of SKOV3 subcutaneous xenograft tumors. **b** Representative images of the excised tumors from the experiment on day 28 after tumor cell injection. **c**, **d** The changes of miR-223 (**c**) and PTEN (**d**) in tumor sections of mice treated as indicated above were plotted on day 28 after tumor cell injection. **e** Representative photographs of PTEN, p-AKT and AKT protein expression of tumors collected from each group detected by western blotting. **f** The immunohistochemistry analyses for PTEN and Ki67 staining were carried out on SKOV3 xenograft tumor sections. Representative staining were shown (× 200 magnification). **g** TUNEL analysis in tumors treated as indicated above (× 50 magnification). *P < 0.05, **P < 0.01

To investigate the effect of TAMs-derived exosomal miR-223 on tumor growth and drug resistance, SKOV3 cells were injected subcutaneously in nu/nu female mice to generate tumors with a size of 60 mm^3^. cDDP and hypoxic or normoxic TAMs exosomes were then injected into the center of the xenograft tumors. Xenografts injected with hypoxic TAMs exosomes or miR-223 overexpression normoxic TAMs exosomes grew statistically significantly larger (Fig. 6a and b) and showed higher miR-223 and lower PTEN expression (Fig. 6c-e). In contrast, miR-223 inhibition abrogated the hypoxic TAMs exosomes-mediated tumor growth in mice. Also, we quantified PTEN and a marker of cell proliferation (Ki67) in tumors. Hypoxic TAMs exosomes and miR-223 expressing normoxic exosomes treatment led to significant increase in the numbers of Ki67-positive cells, reduced PTEN expression, and impaired tumors apoptosis (Fig. 6F-G). A decreased number of Ki67+, downregulation of miR-223, upregulation of PTEN, and increased tumors apoptosis were observed in the xenografts treated with miR-223 inhibiting exosomes (Fig. 6c-g). These observations support that exosomal miR-223 down-regulated PTEN, activated PI3K/AKT signaling, and impaired cDDP therapeutic effects in vivo.

### miR-223 correlates with poor prognosis by targeting PTEN in EOC

**Fig. 7:**
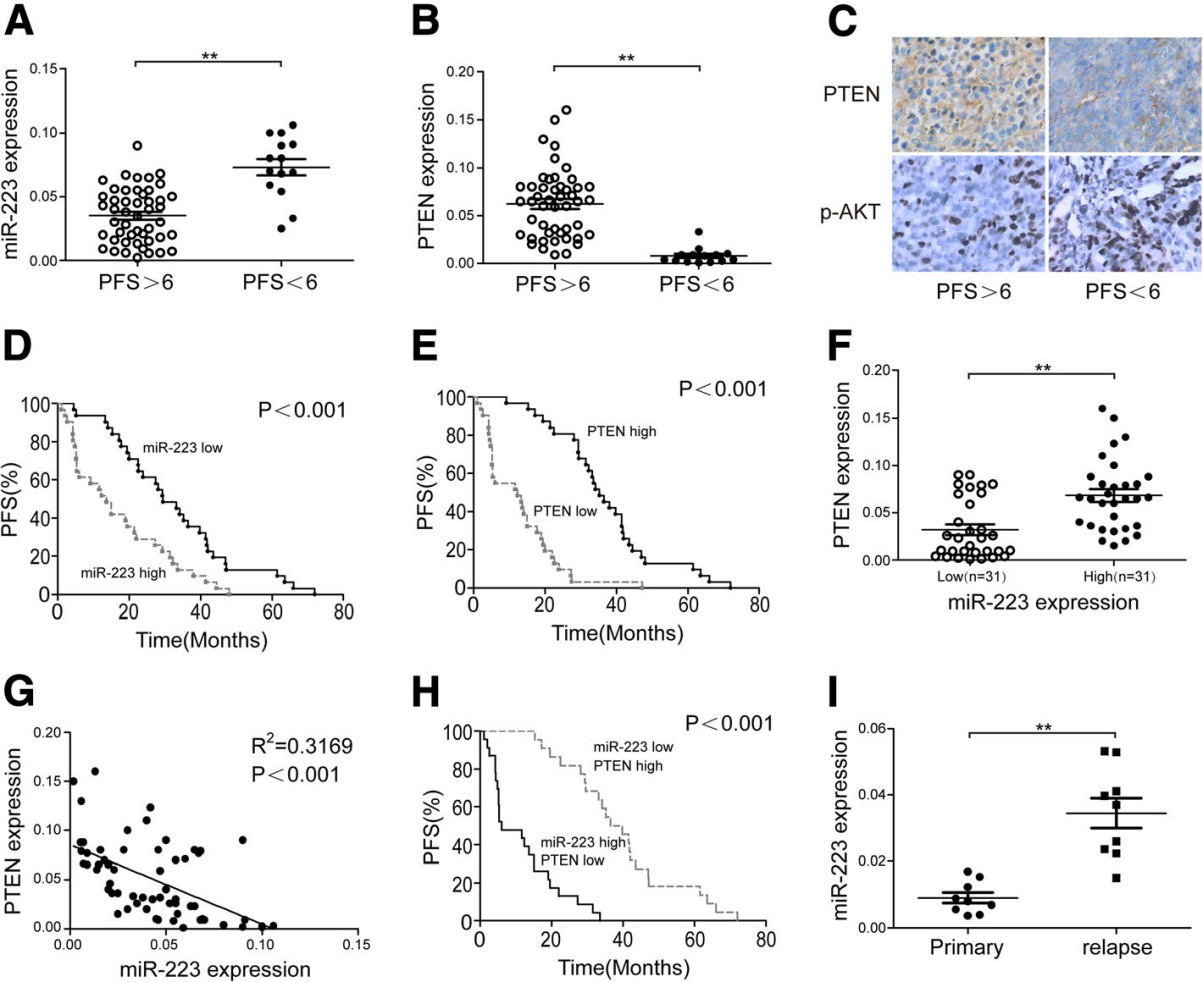
Prognostic role of miR-223 in ovarian cancer patients. The differences of miR-223 (**a**) and PTEN mRNA (**b**) expression between cDDP-sensitive (PFS<6) and –resistant (PFS>6) patients in ovarian cancer specimens was shown. **c** Immunohistochemistry analysis of PTEN and p-AKT. Representative photographs are shown (× 200 magnification). **d**, **e** A Kaplan-Meier analysis of PFS for ovarian cancer patients with the corresponding expression profiles of miR-223 (**d**) and PTEN (**e**) was shown. PTEN and miR-223 expression were inversely correlated in EOC samples (*n* = 62). **f** A plot of the relative expression of miR-223 vs PTEN showed an inverse correlation between them. **g** Correlation index R^2^ were calculated using Spearman rank test (R^2^ = 0.3169). **h** A Kaplan-Meier analysis for PFS of ovarian cancer patients according to anticorrelation miR-223 and PTEN expression. **i** Exosomic miR-223 in 12 EOC patients at the time of primary and recurrent was measured. *P < 0.05, **P < 0.01

Next, we assessed miR-223 and PTEN expression in primary EOC specimens derived from 62 EOC patients. cDDP-resistant patients (PFS<6) showed higher miR-223 expression and lower PTEN expression (Fig. 7a and b). p-AKT protein levels were also elevated in cDDP-resistant patients (Fig. 7c). Similar to high PTEN expression, low miR-223 expression was associated with longer PFS (Fig. 7d and e). Using the median expression value of miR-223 as a cutoff point, the cohort was dichotomized into miR-223-high- or miR-223-low-expressing tumors. PTEN expression was directly and significantly anticorrelated with miR-223 levels (Fig. 7f). By using Spearman’s correlation analysis, an inverse correlation (R^2^ = 0.3169, *P* < 0.001) was validated between miR-223 and PTEN (Fig. 7g), suggesting miR-223-dependent regulation of PTEN. We then split the cohort into four groups by setting PTEN and miR-223 cut-off levels corresponding to low/high mRNA and low/high miRNA expression (each group contains 25% of the patients) for further statistical analysis. High expression of PTEN together with low expression of miR-223 was significantly associated with longer PFS in comparison with low expression of PTEN and high expression levels of miR-223 (Fig. 7h).

To further study the correlation between exosomal miR-223 and the hypoxic tumor microenvironment, the levels of HIF-1a expression in 28 primary EOCs were measured by immunohistochemical staining, and the 28 EOCs were determined and dichotomized into HIF-1a low (*n* = 14) and HIF-1a high (n = 14) (Additional file 8: Figure S6A). Statistically significantly higher intratumoral levels of miR-223, and CD163+ cell infiltration (representative of TAM infiltration) were detected along with higher HIF-1a expression (Additional file 8: Figure S6B and C). These results suggest miR-223 expression and TAM infiltration may be associated with the hypoxic tumor microenvironment.

Further, to analyze the correlation between exosomal miR-223 and recurrence of ovarian cancer, we tested the sera from 12 patients with EOC who received chemotherapy with Taxol and cDDP after first surgery and relapsed within 6 months. We isolated and characterized the serum exosomes, which showed a size distribution consistent with exosome vesicles (50–200 nm) as measured by NTA (Data not shown). These exosomes were highly enriched for the exosome-associated proteins CD63, CD81 and CD9 compared with the serum (Additional file 8: Figure S6D). We then compared the value of miR-223 in exosomes isolated from serum that were collected from EOC patients at the time of their first surgery and recurrence respectively. These patients showed higher levels of circulating exosomal miR-223 at recurrent compared with paired primary EOC patients (Fig. 7i). These results indicate that circulating exosomal miR-223 may correlate with the recurrence of ovarian cancer.

## Discussion

Owing to hypoxic milieu being a persistent feature of TME and a major contributor to malignancy and treatment resistance, understanding the signal regulation and multiple interactions between macrophages and tumor cells under hypoxic microenvironment is of paramount significance [6, 12]. In this study, we investigated whether the paracrine exchange of exosomal miRNAs between EOC cells and neighboring macrophages affected drug resistance.

Macrophages and their precursors can be co-opted by tumors to generate a supportive stroma [5, 30]. Depletion of TAMs by clodronate-loaded liposomes or a CSF-1R inhibitor augmented the effect of anti-cancer treatment [31]. The well described entrapment of macrophages to hypoxic necrotic region could also affect macrophage infiltration and macrophage M2 polarization through an alternative mechanism involving signals emanating from hypoxic tumor cells [32], we found hypoxic EOC cells recruited macrophages and induced their M2 polarization, and the recruited TAMs may be trapped by hypoxic EOC cells. Consistently, a significant amount of TAMs were detected in the hypoxic region of clinical tumor specimens from EOC patients.

Previous study revealed the number of exosomes isolated from ovarian cancer patient sera was 5-fold higher than that from patients with benign disease [33]. Hypoxia has been described as a regulator of MV formation and shedding [34, 35]. The reasons may come from that HIF mediates the induction of Rab22 and RAB20 [36–38], and hypoxia may induce ceramide production by the activation of nSMase, which all may be associated with extracellular vesicle formation and secretion [39]. Here, we show hypoxia increased the release of exosomes from donor cells (TAMs) and also increased the absorption of exosomes by recipient cells (EOC cells).

By means of exosomes, TAMs could render recipient EOC cells much more aggressive under hypoxic than normoxic conditions. Further studies showed hypoxic TAMs derived exosomes promoted chemoresistance of recipient EOC cells via miR-223 transmission, and the ability of miR-223 to enhance chemoresistance was also consistent with a previous study showing miR-223 increased gastric cancer cell resistance [40, 41], and that miR-223 was the most up-regulated in recurrent cancers when compared to primary [18], we showed that circulating miR-223 levels were increased in the serum exosomes of recurrent EOC patients. Our model also suggested that high levels of miR-223 frequently observed in homogenates of primary tumors might not be due to the high miR-223 expression in cancer cells, but because of the exosomal transfer of miR-223 from the surrounding TAMs.

C/EBPα and PU.1 were confirmed to be able to up-regulate the level of miR-223 [42, 43], combined with previous studies showing that HIF-1α can enhance the transcriptional activity of C/EBPα in a transcription-independent manner [44]. We showed that pharmacological inducer or inhibitor of HIF-1α could significantly increase or decrease the expression of miR-223 respectively. Consistently, the high expression of HIF-1α was positively associated with intratumoral levels of miR-223. However, the molecular mechanism remains to be further explored.

Specifically, TAMs transferred exosomal miR-223 was able to directly target PTEN, a protein that is involved in the regulation of drug resistance of EOC and other cancer cells [45, 46]. PTEN was known to repress cell survival signaling, such as AKT, and thereby to promote cell death [46, 47]. In our experiments, miR-223-mediated PTEN reduction led to increased PI3K/AKT signal activation over time, suggesting AKT may be involved in miR-223/PTEN pathway. Finally, the negative correlation between miR-223 and PTEN were observed in different types of cancer cells, suggesting a common mechanism existed in different cancers.

However, we only focused on miR-223, which had higher level in exosomes compared with cells, particularly highly expressed in macrophages and had important regulatory roles in drug resistance [17, 18]. In our results, miR-223 was of low levels in EOC cells (Additional file 6: Figure S4B), but got up-regulated after coculture with macrophages under hypoxic conditions. Although the differences were statistically significant, inhibition of miR-223 expression does not completely abolish the promotion of chemoresistance by exosomes produced by TAMs. The reason might be that there are still other factors, besides miR-223, including the protein cargo of exosomes and other transferred miRNAs between cells contributing to the exosome-induced chemoresistance, which needs to be explored in the future. Moreover, except for PTEN, other targets might also be modulated by TAM derived exosomal miR-223 in EOC cells. Future studies on the above-mentioned aspects are suggested.

## Conclusions

In summary, we provided evidence that hypoxic EOC cells induced macrophage M2-polarization, and hypoxia up-regulated the miR-223 levels in TAMs-derived exosomes; these miR-223-rich TAMs exosomes were then internalized by EOC cells and induced the recipient cells towards a chemoresistance phenotype. Thus, we identified a new exosomal miR-223/PTEN/PI3K/AKT axis triggered drug resistance in EOC cells, and verified exosomes within the TME as important molecular targets to restore drug sensitivity. Moreover, circulating exosomal miR-223 may function as a biomarker for predicting the response to chemotherapy of patients with advanced EOC. The knowledge of these cancer cell-secreted molecules and their role in tumor microenvironment might permit a better understanding on the biology of tumor, and the identification of novel potential serum biomarkers of drug response or novel drug targets to overcome EOC chemoresistance.

## Additional files

Additional file 1: Supplementary Methods. (DOC 47 kb)

Additional file 2: **Table S1.** Clinicopathological characteristics of EOC patients. (*n* = 62) (DOC 36 kb)

Additional file 3: **Figure S1.** (A) CD163 positive rate in macrophages cocultured with EOC cells under normoxia. (B) The related fold change of CD206, Arg-1 and MCP-1 mRNA levels in macrophages alone or cocultured with normoxic or hypoxic A2780 cells for 48 h. (C) IL-10 and IL-12 expression levels in macrophages evaluated by ELISA. *P < 0.05, **P < 0.01 (TIF 342 kb)

Additional file 4: **Figure S2.** Exosome uptake ratio of SKOV3 (A) and A2780 (B) cocultured with macrophages under normoxia and hypoxia. *P < 0.05, **P < 0.01 (TIF 1357 kb)

Additional file 5: **Figure S3.** TAMs derived exosomes confer drug resistance of recipient EOC cells. A2780 cells were treated with PBS, N-Mcr-exo or H-Mcr-exo for 24 h, cell apoptosis (A), cell viability (B), was measured in A2780 cells respectively. (C-D) A2780 cells were cultured in TAMs-conditioned or exosome-depleted TAMs-conditioned media, cell apoptosis (C) cell viability (D), IC50 for cDDP (E) was analyzed in A2780 cells. A2780 cells were treated with DMSO or GW4869, cell apoptosis (F), cell viability (G, H), IC50 for cDDP (I) was detected. *P < 0.05, **P < 0.01 (TIF 1036 kb)

Additional file 6: **Figure S4.** Exosomic miR-223 mediated cross-talk between macrophages and EOC cells. (A) Related miR-223 level in macrophages under normoxia and hypoxia. (B) Related miR-223 level in normoxic or hypoxic macrophages derived exosomes. (C) Fold change of miR-223 in A2780 cells cocultured with macrophages for the indicated time periods under normoxia or hypoxia. (D) Related miR-223 level in A2780 cells treated with PBS, N-Mcr-exo or H-Mcr-exo. (E) Related pre-miR-223 levels in EOC cells cultured alone, cocultured with macrophages or treated with H-Mcr-exo. (F-G) Fold change of miR-223 in A2780 cells cocultured with TAMs-conditioned, exosome-depleted TAMs-conditioned media or TAMs-conditioned media pretreated with GW4869 under normoxia (F) and hypoxia (G). (H) Fold change of miR-223 in A2780 treated with RNA polymerase II inhibitor or PBS. (I-J) Related miR-223 level in macrophages (I) or exosomes derived from macrophages (J) treated with PBS, DMOG or YC-1 under normoxia and hypoxia. (K-L) A2780 cells were treated with exosomes derived from the normoxic or hypoxic macrophages which were transfected with agomir or antagomir respectively, the related miR-223 level was measured. (M) The number of cell colonies was detected in SKOV3 treated as indicated. *P < 0.05, **P < 0.01 (TIF 2315 kb)

Additional file 7: **Figure S5.** (A-B) Related miR-223 (A) and PTEN protein (B) level in MCF-7, MBA-MD-231 and Hela cells treated with PBS or H-Mcr-exo, (C) IC50 for cDDP was measured in SKOV3 cells (pretreated with LY294002) cocultured with exosomes derived from the normoxic or hypoxic macrophages which were transfected with agomir. *P < 0.05, **P < 0.01 (TIF 395 kb)

Additional file 8: **Figure S6.** (A) Representative images of low (left) or high (right) HIF-1a expression in EOC samples by immunohistochemical staining. (magnification, × 200). (B-C) Intertumoral level of miR-223 (B), and CD163+ cell infiltration (C) (representative of TAMs infiltration) were measured with high and low HIF-1a expression in 28 primary EOC tissues. (D) Representative images of CD81, CD63, and CD9 in serum and its derived exosomes from an EOC patient. *P < 0.05, **P < 0.01 (TIF 1007 kb)

## Abbreviations

HIF-1: Hypoxia inducible factor-1
PI3K: Phosphatidylinositol 3-kinase
PTEN: Gene of phosphate and tension homology deleted on chromosome ten
TAM: Tumor-associated macrophage

## References

[1] Liu D, Zhang XX, Li MC, Cao CH, Wan DY, Xi BX (2018). C/EBPbeta enhances platinum resistance of ovarian cancer cells by reprogramming H3K79 methylation. Nat Commun.

[2] Koelwyn GJ, Quail DF, Zhang X, White RM, Jones LW (2017). Exercise-dependent regulation of the tumour microenvironment. Nat Rev Cancer.

[3] Welford AF, Biziato D, Coffelt SB, Nucera S, Fisher M, Pucci F (2011). TIE2-expressing macrophages limit the therapeutic efficacy of the vascular-disrupting agent combretastatin A4 phosphate in mice. J Clin Invest.

[4] Brown JM, Recht L, Strober S (2017). The promise of targeting macrophages in Cancer therapy. Clin Cancer Res.

[5] Squadrito ML, Etzrodt M, De Palma M, Pittet MJ (2013). MicroRNA-mediated control of macrophages and its implications for cancer. Trends Immunol.

[6] Wen Z, Liu H, Li M, Li B, Gao W, Shao Q (2015). Increased metabolites of 5-lipoxygenase from hypoxic ovarian cancer cells promote tumor-associated macrophage infiltration. Oncogene.

[7] DeNardo DG, Brennan DJ, Rexhepaj E, Ruffell B, Shiao SL, Madden SF (2011). Leukocyte complexity predicts breast cancer survival and functionally regulates response to chemotherapy. Cancer Discov.

[8] Na YR, Je S, Seok SH (2018). Metabolic features of macrophages in inflammatory diseases and cancer. Cancer Lett.

[9] Song M, Liu T, Shi C, Zhang X, Chen X (2016). Bioconjugated manganese dioxide nanoparticles enhance chemotherapy response by priming tumor-associated macrophages toward M1-like phenotype and attenuating tumor hypoxia. ACS Nano.

[10] Yogev O, Henderson S, Hayes MJ, Marelli SS, Ofir-Birin Y, Regev-Rudzki N (2017). Herpesviruses shape tumour microenvironment through exosomal transfer of viral microRNAs. PLoS Pathog.

[11] Pucci F, Pittet MJ (2013). Molecular pathways: tumor-derived microvesicles and their interactions with immune cells in vivo. Clin Cancer Res.

[12] King HW, Michael MZ, Gleadle JM (2012). Hypoxic enhancement of exosome release by breast cancer cells. BMC Cancer.

[13] Kucharzewska P, Christianson HC, Welch JE, Svensson KJ, Fredlund E, Ringner M, Morgelin M, Bourseau-Guilmain E, Bengzon J, Belting M (2013). Exosomes reflect the hypoxic status of glioma cells and mediate hypoxia-dependent activation of vascular cells during tumor development. Proc Natl Acad Sci U S A.

[14] Dorayappan KDP, Wallbillich JJ, Cohn DE, Selvendiran K (2016). The biological significance and clinical applications of exosomes in ovarian cancer. Gynecol Oncol.

[15] Dorayappan KDP, Wanner R, Wallbillich JJ, Saini U, Zingarelli R, Suarez AA (2018). Hypoxia-induced exosomes contribute to a more aggressive and chemoresistant ovarian cancer phenotype: a novel mechanism linking STAT3/Rab proteins. Oncogene.

[16] Shurtleff MJ, Temoche-Diaz MM, Karfilis KV, Ri S, Schekman R. Y-box protein 1 is required to sort microRNAs into exosomes in cells and in a cell-free reaction. eLife.2016;5: pii: e19276. 10.7554/eLife.19276 PMC5047747 27559612

[17] Aucher A, Rudnicka D, Davis DM (2013). MicroRNAs transfer from human macrophages to hepato-carcinoma cells and inhibit proliferation. J Immunol.

[18] Laios A, O'Toole S, Flavin R, Martin C, Kelly L, Ring M (2008). Potential role of miR-9 and miR-223 in recurrent ovarian cancer. Mol Cancer.

[19] de Melo Maia B, Rodrigues IS, Akagi EM, Soares do Amaral N, Ling H, Monroig P (2016). MiR-223-5p works as an oncomiR in vulvar carcinoma by TP63 suppression. Oncotarget.

[20] Singh M, Chaudhry P, Fabi F, Asselin E (2013). Cisplatin-induced caspase activation mediates PTEN cleavage in ovarian cancer cells: a potential mechanism of chemoresistance. BMC Cancer.

[21] Frankson R, Yu ZH, Bai Y, Li Q, Zhang RY, Zhang ZY (2017). Therapeutic targeting of oncogenic tyrosine phosphatases. Cancer Res.

[22] Xia H, Ooi LL, Hui KM (2013). MicroRNA-216a/217-induced epithelial-mesenchymal transition targets PTEN and SMAD7 to promote drug resistance and recurrence of liver cancer. Hepatology.

[23] Zhu X, Shen H, Yin X, Long L, Xie C, Liu Y (2016). miR-186 regulation of Twist1 and ovarian cancer sensitivity to cisplatin. Oncogene.

[24] O'Connell RM, Taganov KD, Boldin MP, Cheng G, Baltimore D (2007). MicroRNA-155 is induced during the macrophage inflammatory response. Proc Natl Acad Sci U S A.

[25] Murdoch C, Giannoudis A, Lewis CE (2004). Mechanisms regulating the recruitment of macrophages into hypoxic areas of tumors and other ischemic tissues. Blood.

[26] Anderson JD, Johansson HJ, Graham CS, Vesterlund M, Pham MT, Bramlett CS (2016). Comprehensive proteomic analysis of mesenchymal stem cell exosomes reveals modulation of angiogenesis via nuclear factor-KappaB signaling. Stem Cells.

[27] Hsu YL, Hung JY, Chang WA, Lin YS, Pan YC, Tsai PH (2017). Hypoxic lung cancer-secreted exosomal miR-23a increased angiogenesis and vascular permeability by targeting prolyl hydroxylase and tight junction protein ZO-1. Oncogene.

[28] Valadi H, Ekstrom K, Bossios A, Sjostrand M, Lee JJ, Lotvall JO (2007). Exosome-mediated transfer of mRNAs and microRNAs is a novel mechanism of genetic exchange between cells. Nat Cell Biol.

[29] Semenza GL (2012). Hypoxia-inducible factors in physiology and medicine. Cell.

[30] Zhu C, Mustafa D, Zheng PP, van der Weiden M, Sacchetti A, Brandt M (2017). Activation of CECR1 in M2-like TAMs promotes paracrine stimulation-mediated glial tumor progression. Neuro-Oncology.

[31] Priceman SJ, Sung JL, Shaposhnik Z, Burton JB, Torres-Collado AX, Moughon DL (2010). Targeting distinct tumor-infiltrating myeloid cells by inhibiting CSF-1 receptor: combating tumor evasion of antiangiogenic therapy. Blood.

[32] Tripathi C, Tewari BN, Kanchan RK, Baghel KS, Nautiyal N, Shrivastava R (2014). Macrophages are recruited to hypoxic tumor areas and acquire a pro-angiogenic M2-polarized phenotype via hypoxic cancer cell derived cytokines Oncostatin M and Eotaxin. Oncotarget.

[33] Gercel-Taylor C, Atay S, Tullis RH, Kesimer M, Taylor DD (2012). Nanoparticle analysis of circulating cell-derived vesicles in ovarian cancer patients. Anal Biochem.

[34] Zhang W, Zhou X, Yao Q, Liu Y, Zhang H, Dong Z (2017). HIF-1-mediated production of exosomes during hypoxia is protective in renal tubular cells. Am J Physiol Renal Physiol.

[35] Steinbichler TB, Dudas J, Riechelmann H, Skvortsova II (2017). The role of exosomes in cancer metastasis. Semin Cancer Biol.

[36] Hackenbeck T, Huber R, Schietke R, Knaup KX, Monti J, Wu X (2011). The GTPase RAB20 is a HIF target with mitochondrial localization mediating apoptosis in hypoxia. Biochim Biophys Acta.

[37] Wang T, Gilkes DM, Takano N, Xiang L, Luo W, Bishop CJ (2014). Hypoxia-inducible factors and RAB22A mediate formation of microvesicles that stimulate breast cancer invasion and metastasis. Proc Natl Acad Sci U S A.

[38] Colombo M, Raposo G, Thery C (2014). Biogenesis, secretion, and intercellular interactions of exosomes and other extracellular vesicles. Annu Rev Cell Dev Biol.

[39] Kosaka N, Iguchi H, Hagiwara K, Yoshioka Y, Takeshita F, Ochiya T (2013). Neutral sphingomyelinase 2 (nSMase2)-dependent exosomal transfer of angiogenic microRNAs regulate cancer cell metastasis. J Biol Chem.

[40] Eto K, Iwatsuki M, Watanabe M, Ishimoto T, Ida S, Imamura Y (2015). The sensitivity of gastric cancer to trastuzumab is regulated by the miR-223/FBXW7 pathway. Int J Cancer.

[41] Zhou X, Jin W, Jia H, Yan J, Zhang G (2015). MiR-223 promotes the cisplatin resistance of human gastric cancer cells via regulating cell cycle by targeting FBXW7. J Exp Clin Canc Res : CR.

[42] Fazi F, Rosa A, Fatica A, Gelmetti V, De Marchis ML, Nervi C (2005). A minicircuitry comprised of microRNA-223 and transcription factors NFI-A and C/EBPalpha regulates human granulopoiesis. Cell.

[43] Fukao T, Fukuda Y, Kiga K, Sharif J, Hino K, Enomoto Y (2007). An evolutionarily conserved mechanism for microRNA-223 expression revealed by microRNA gene profiling. Cell.

[44] Zhang J, Chen GQ (2009). Hypoxia-HIF-1alpha-C/EBPalpha/Runx1 signaling in leukemic cell differentiation. Pathophysiology.

[45] Chen Q, Qin R, Fang Y, Li H (2015). Berberine sensitizes human ovarian Cancer cells to cisplatin through miR-93/PTEN/Akt signaling pathway. Cell Physiol Biochem.

[46] Keniry M, Parsons R (2008). The role of PTEN signaling perturbations in cancer and in targeted therapy. Oncogene.

[47] Cowell JK, Qin H, Hu T, Wu Q, Bhole A, Ren M (2017). Mutation in the FGFR1 tyrosine kinase domain or inactivation of PTEN is associated with acquired resistance to FGFR inhibitors in FGFR1-driven leukemia/lymphomas. Int J Cancer.

