# Supplementary material for: Macrophages derived exosomes deliver miR-223 to epithelial ovarian cancer cells to elicit a chemoresistant phenotype
Source: J Exp Clin Cancer Res. 2019 Feb 15;38:81. doi: 10.1186/s13046-019-1095-1 (PMC6377760; doi:10.1186/s13046-019-1095-1)
Supplement: Supplementary file 1 — Supplementary Methods. (DOC 47 kb) [file 13046_2019_1095_MOESM1_ESM.doc]

**Additional file 1: Supplementary Methods**

**Cell culture and treatment**

Cells were cultured in DMEM supplemented with 10% fetal calf serum (Gibco BRL, Grand Island, NY), 100 U/mL penicillin and 100 µg/mL streptomycin in a humidified atmosphere containing 5% CO_2_ at 37°C.

# For preparation of M2-polarized THP-1 macrophages, 100 ng/ml phorbol 12-myristate 13-acetate (PMA, ab120297, Abcom, USA) was added to THP-1 cells for 6 hrs, followed by PMA plus 20 ng/ml interleukin (IL)-4 (Sigma Aldrich, USA) for the following 18 hrs. After three washes to remove cytokines, M2-polarized THP-1 macrophages were cocultured in transwell inserts (3540, Corning) with EOC cells in 6-well plates (1×10^5^ cells/well) without direct contact.

# For hypoxic conditions (1% oxygen), cells were cultured in a modular incubator chamber (Billups-Rothenburg, Del Mar, CA) that had been flushed with a mixture of O_2_, N_2_ (the ratio was depended by the deigned proposal separately) and 5% CO_2_. DMOG and YC-1 were purchased from Selleck in USA.

**Exosome collection, characterization and quantification**

Collection: A total of 250 μL serum (which was separated by centrifugation and frozen at - 80 °C) or 20 mL of tissue culture media was mixed with ExoQuick exosome precipitation solution, and exosome isolation was performed according to the manufacturer's instructions (SBI System Biosciences).

Exosome preparations were verified by Transmission electron microscopy (TEM). Exosomes were dissolved in PBS buffer, dropped into a carbon-coated copper grid and then were stained with 2% uranyl acetate. Images of the sample were captured using a Tecnai G2 Spirit electron microscope (FEI Co., Hillsboro, OR, USA). The size distribution and concentration of the exosomes were detected by Nanoparticle Tracking Analysis (NTA) with ZetaView (Particle Metrix Inc, Germany). Isolated exosomes were diluted with 1:500 or 1:1000 in particle-free PBS and resuspended before being injected into the sample cell chamber. Size distributions and particle concentrations were assessed with NTA software. Exosome concentration analysis was normalized with the total number of cells from the corresponding dish. To quantify the cell number, the cells in each dish were harvested at the end of treatment and digested into suspension by trypsin for quantification with a TC20 Automated Cell Counter.

**Exosome labeling and tracking**

Isolated exosomes from the supernatant of macrophages were labeled using a PKH67 green fluorescent cell linker kit (Sigma Aldrich, USA) according to the manufacture’s protocol. Labeled exosomes were cocultured with EOC cells for 24 hrs and were washed off subsequently. The uptake of labeled exosomes by recipient cells was detected by a Leica TCS SP5 II laser scanning confocal microscope.

**Transfection**

Macrophages were seeded into 6-well plates (6×10^5^ cells/well) and transfected with miR-223 agomir, antagomir or the corresponding negative control (agomir-NC, antagomir-NC) (GenePharma, Shanghai, China) using HiperFect transfection reagent (Qiagen GmbH, Hilden, Germany). The recombinant plasmid eukaryotic expression vector pcDNA3.1-PTEN (PTEN) and the empty vector control (vector) were purchased from Cell Signaling Technology, Inc. (Beverly, USA). PTEN was transfected into EOC cells (6×10^5^ cells/well) using Lipofectamine 2000 according to the manufacturer’s protocol.

**Reverse transcription quantitative real-time PCR**

Total RNA was extracted with TRIzol reagent (Invitrogen, Carlsbad, CA). Complementary DNA (cDNA) was synthesized using a PrimeScript RT reagent Kit (TaKaRa, Dalian, China). Real-time polymerase chain reaction (PCR) analyses were performed with SYBR Premix Ex Taq (TaKaRa), and the 2^-ΔΔCT^ method was used to calculate gene expression. The relative levels of miRNA and mRNA were normalized to U6 snRNA and β-actin, respectively. The primers used are listed in Supplementary Methods.

Primers for reverse transcription quantitative real-time PCR

|  | Forward | Reverse |
| --- | --- | --- |
| CD206 | 5′-GGGTTGCTATCACTCTCTATGC-3′ | 5′-TTTCTTGTCTGTTGCCGTAGTT-3′ |
| Arg-1 | 5′-TGGACAGACTAGGAATTGGCA-3′ | 5′–CCAGTCCGTCAACATCAAAACT-3′ |
| MCP-1 | 5′–CAGCCAGATGCAATCAATGCC-3′ | 5′–TGGAATCCTGAACCCACTTCT-3′ |
| β-actin | 5′–CCTGGCACCCAGCACAAT-3′ | 5′–GGGCCGGACTCGTCATACT-3′ |

**Western blot analysis**

# Proteins (40-50mg) from cells or tissues were separated by SDS-PAGE and then transferred to polyvinylidene difluoride membranes (PVDF; Bio-Rad, USA). The membranes were blocked and then probed with antibodies against PTEN (1:100; ab79156), p-AKT (1:2000; ab81283), HIF-1α (1:2000; ab16066) from Abcam, Cambridge, MA, USA. CD63 (1:100; sc-15363), CD81 (1:100; sc-70803), CD9 (1:100; sc-9148), Alix (1:200; sc-53540) were acquired from Santa Cruz Biotechnology (Santa Cruz, CA, USA), or β-actin (1:5000; A9044, Sigma, St. Louis, MO). After washing, the blots were incubated with horseradish peroxidase-conjugated secondary antibodies.

**Luciferase reporter assays**

The putative target sites of the human PTEN 3’UTR segments for miR-223 were amplified using Pyrobest DNA polymerase (Fermentas, CA) and then cloned into the Xba I site of the pGL3 control plasmid (Promega, Madison, USA). pRL-TK Vector expressing renilla luciferase was used as control vector. miR-223 binding sites in the PTEN 3’UTR were amplified by PCR. The respective mutated putative miR-223 binding site in the PTEN 3’UTR (Mut-PTEN 3’UTR) were generated using the Quick change site-directed mutagenesis kit (Stratagene, Cedar Creek, USA) according to the manufacturer’s protocol. For reporter assays, EOC cells were transiently cotransfected with pRL-TK Vector (Promega), wild-type or mutant 3’UTR of PTEN and agomir or agomir-NC by lipofectamine 2000, firefly and renilla luciferase activities were measured consecutively using Dual-Luciferase Reporter assay system (Promega). The luminescence intensity of firefly luciferase was normalized to that of renilla luciferase.

**Flow cytometric analysis**

The detailed procedures and materials are described in the Supplementary Methods.

# The primary human macrophages were recovered from coculture or differentiation experiments and stained in flow buffer (PBS with 1% BSA and 0.1% NaN3). The antibodies used were: APC anti-human CD163 (Biolegend, San Diego, CA). Samples were acquired and analyzed with an LSRII flow cytometer (BD Biosciences, San Jose, CA).

**Evaluation of apoptosis**

After transfection, EOC cells were treated with cDDP for 24 hrs. Cell death was evaluated with Annexin-V-FITC and propidium iodide (PI) double staining using a dead cell apoptosis kit according to the manufacturer’s protocol (556547, Annexin V-FITC Apoptosis Detection Kit I, BD Biosciences, San Jose, CA, USA). Cells positive for Annexin-V and/or PI were considered dead. Terminal deoxynucleotidyl transferase dUTP nick end labeling (TUNEL) (In situ cell death detection kit, Fluorescein, 11684795910, Roche, Basel, Switzerland) was used to assess apoptosis according to the instructions.

**Assessing chemosensitivity to cDDP**

Cells were plated into 96-well plates (5×10^3^ cells/well) and exposed to various doses of cDDP (0.01, 0.05, 0.25, 1.25, 7.5 and 25µM) for 48 hrs. Then 10μl of CCK-8 solution was added to each well, and the plate was incubated for 3 hrs in a humidified incubator. Then the absorbance of each well was measured at 450 nm using a Model 550 series microplate reader (Bio-Rad Laboratories). Cell viability was expressed as the ratio of the treated cells to that of the untreated controls at each dose or concentration. The IC50 value for each cell line was determined by nonlinear regression analysis using GraphPad Prism (GraphPad Software Inc., San Diego, CA). For colony formation, after transfection, around 500 SKOV3 cells were seeded in each 6-cm dish and treated with 1.25µM cDDP 6 h after seeding. After 8-10 days, colonies were fixed and stained with 0.3% crystal violet in methanol, and then the number of colonies was counted manually.

**Cell migration assays**

For migration assays, macrophages were harvested and re-suspended in serum-free DMEM medium then were deposited onto an 8 mm pore size polycarbonate membrane Transwell insert (Corning Costar, MD, USA). The conditioned medium of normoxic or hypoxic EOC cells was added into the lower chamber. After 24 hrs of incubation at 37℃, cells adhering to the lower membrane were stained with 0.1% crystal violet in 20% methanol, imaged, and counted using an IX71 inverted microscope (Olympus, Tokyo, Japan).

# Immunohistochemistry and scoring

# Tumors were fixed, embedded in paraffins and sectioned into 4-μm thick. After deparaffinization and rehydration, sections were blocked and then incubated with antibodies against PTEN (ab79156), p-AKT (ab81283), HIF-1α (ab16066), CD163 (sc-20066), Ki-67 (sc-56320), and then detected using the Dako Envision two-step method of immunohistochemistry (Carpinteria, CA, USA). All IHC staining was scored independently by two pathologists. Nuclear HIF-1α, KI-67 and cytoplasmic CD163 expressions were interpreted. We scored the positive staining results in categories from 0 to 3+ as follows: 0, no staining; 1+, 1-25% of the specimen stained; 2+, 26-50%; and 3+, >50%. A score of 3+ was considered to be a positive IHC result.
