# Supplementary material for: Macrophages derived exosomes deliver miR-223 to epithelial ovarian cancer cells to elicit a chemoresistant phenotype
Source: J Exp Clin Cancer Res. 2019 Feb 15;38:81. doi: 10.1186/s13046-019-1095-1 (PMC6377760; doi:10.1186/s13046-019-1095-1)
Supplement: Supplementary file 2 — Table S1. Clinicopathological characteristics of EOC patients. (n = 62) (DOC 36 kb) [file 13046_2019_1095_MOESM2_ESM.doc]

Additional file 2: Table S1. Clinicopathological characteristics of EOC patients with either high or low miR-223 expression. (n= 62)

|  | | miR-223 low | miR-223 high |
| --- | --- | --- | --- |
| Age（yrs） |  | 54.21±8.29 | 55.31±7.93 |
| Stage | IIIC | 24(38.71%) | 26 (41.94%) |
|  | Ⅳ | 5(8.06%) | 7(11.29%) |
| Grade | High | 3(4.84%) | 2(3.23%) |
|  | Moderate | 9(14.52%) | 8(12.90%) |
|  | Low | 21 (33.87%) | 19(30.65%) |
