# Supplementary figures and images for: Macrophages derived exosomes deliver miR-223 to epithelial ovarian cancer cells to elicit a chemoresistant phenotype
Source: J Exp Clin Cancer Res. 2019 Feb 15;38:81. doi: 10.1186/s13046-019-1095-1 (PMC6377760; doi:10.1186/s13046-019-1095-1)

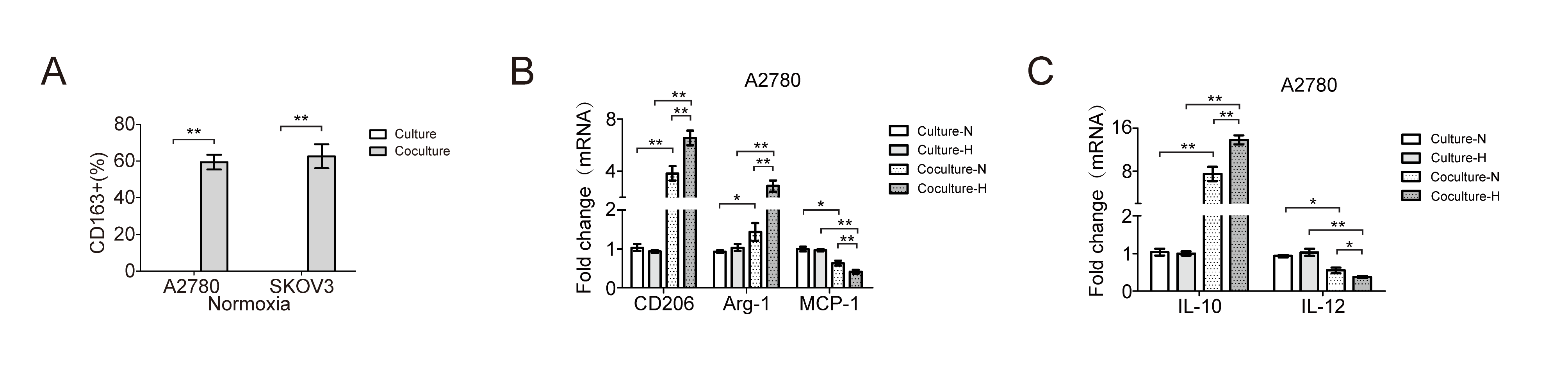

Supplement: Supplementary file 3 — Figure S1. (A) CD163 positive rate in macrophages cocultured with EOC cells under normoxia. (B) The related fold change of CD206, Arg-1 and MCP-1 mRNA levels in macrophages alone or cocultured with normoxic or hypoxic A2780 cells for 48 h. (C) IL-10 and IL-12 expression levels in macrophages evaluated by ELISA. *P < 0.05, **P < 0.01 (TIF 342 kb) [file 13046_2019_1095_MOESM3_ESM.tif]

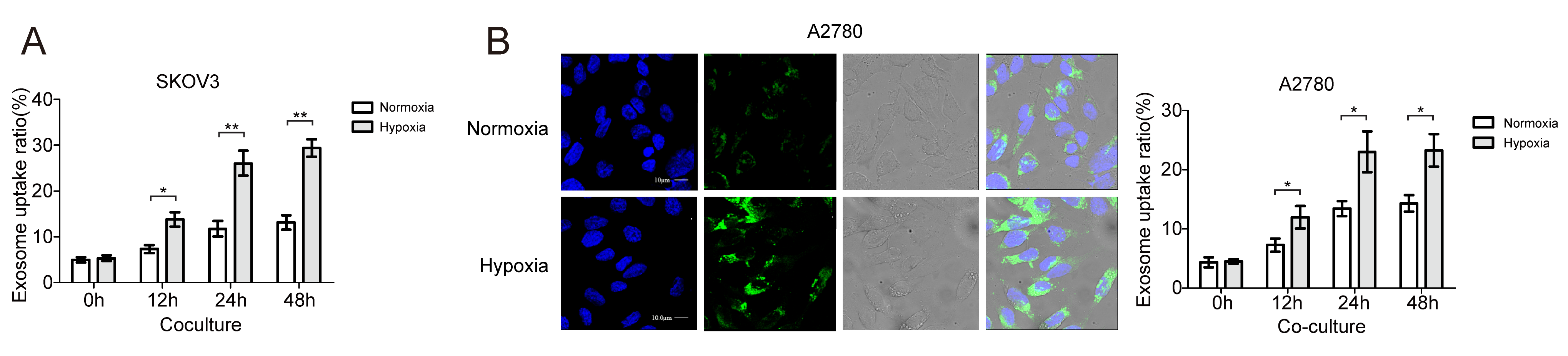

Supplement: Supplementary file 4 — Figure S2. Exosome uptake ratio of SKOV3 (A) and A2780 (B) cocultured with macrophages under normoxia and hypoxia. *P < 0.05, **P < 0.01 (TIF 1357 kb) [file 13046_2019_1095_MOESM4_ESM.tif]

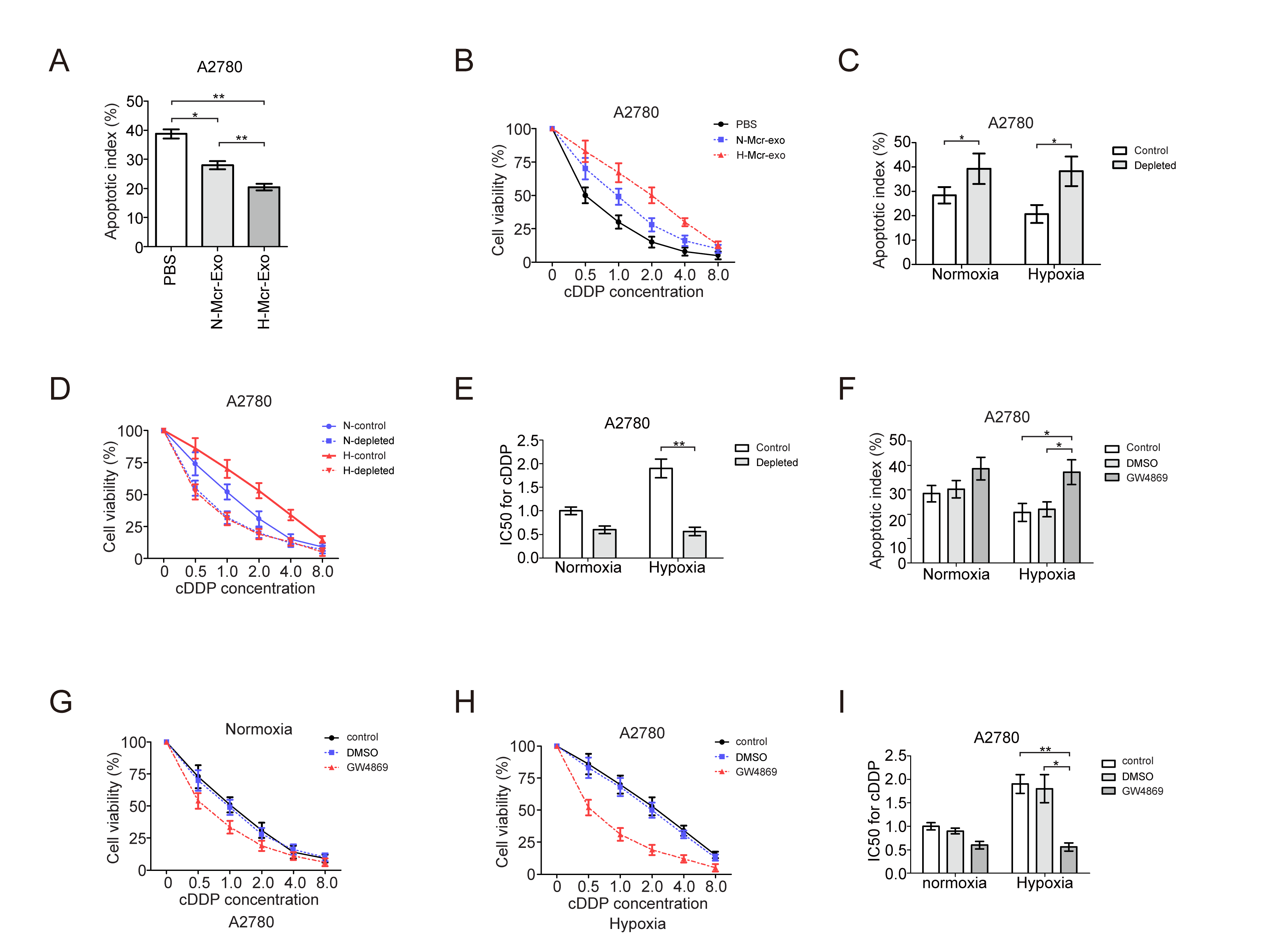

Supplement: Supplementary file 5 — Figure S3. TAMs derived exosomes confer drug resistance of recipient EOC cells. A2780 cells were treated with PBS, N-Mcr-exo or H-Mcr-exo for 24 h, cell apoptosis (A), cell viability (B), was measured in A2780 cells respectively. (C-D) A2780 cells were cultured in TAMs-conditioned or exosome-depleted TAMs-conditioned media, cell apoptosis (C) cell viability (D), IC50 for cDDP (E) was analyzed in A2780 cells. A2780 cells were treated with DMSO or GW4869, cell apoptosis (F), cell viability (G, H), IC50 for cDDP (I) was detected. *P < 0.05, **P < 0.01 (TIF 1036 kb) [file 13046_2019_1095_MOESM5_ESM.tif]

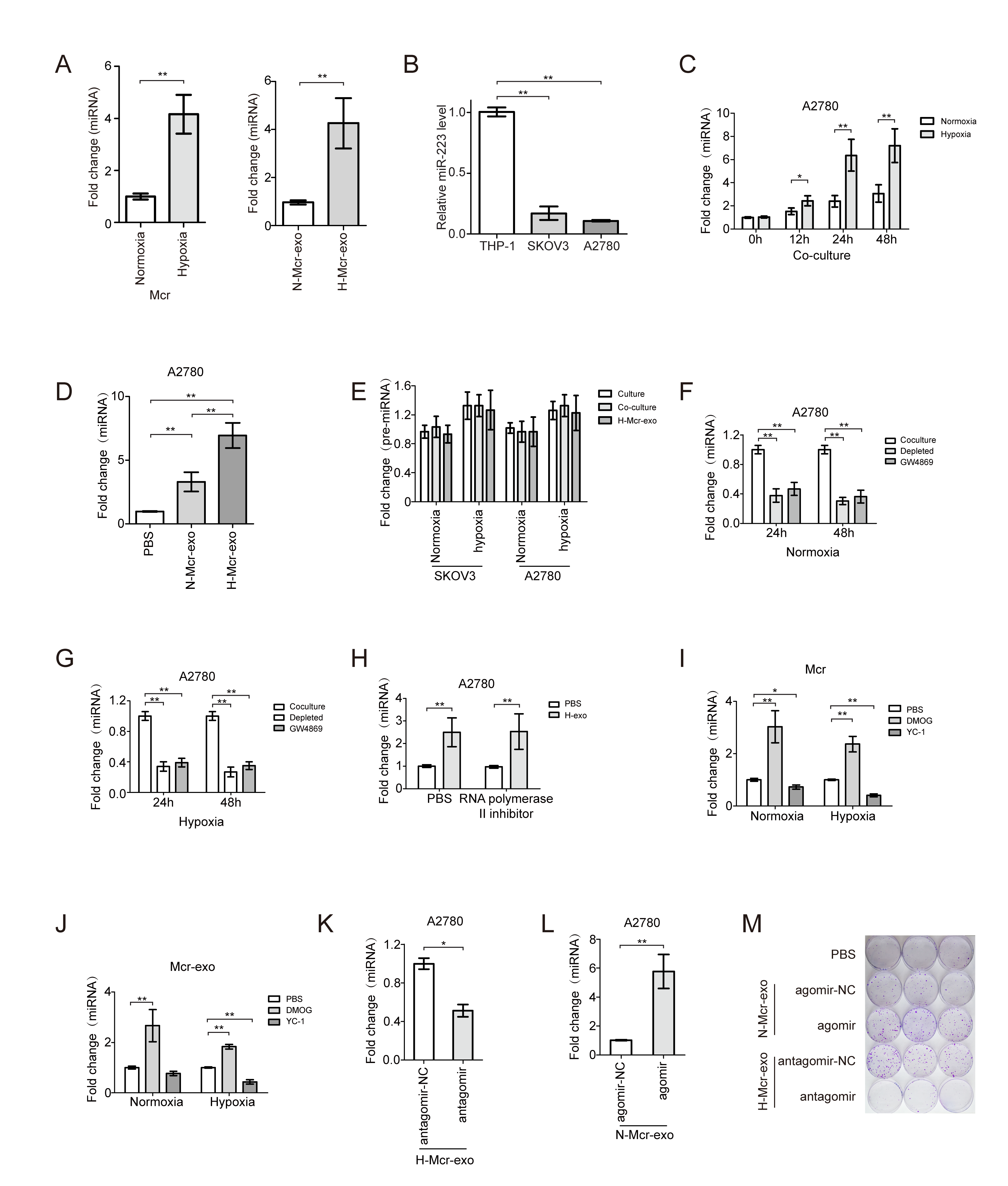

Supplement: Supplementary file 6 — Figure S4. Exosomic miR-223 mediated cross-talk between macrophages and EOC cells. (A) Related miR-223 level in macrophages under normoxia and hypoxia. (B) Related miR-223 level in normoxic or hypoxic macrophages derived exosomes. (C) Fold change of miR-223 in A2780 cells cocultured with macrophages for the indicated time periods under normoxia or hypoxia. (D) Related miR-223 level in A2780 cells treated with PBS, N-Mcr-exo or H-Mcr-exo. (E) Related pre-miR-223 levels in EOC cells cultured alone, cocultured with macrophages or treated with H-Mcr-exo. (F-G) Fold change of miR-223 in A2780 cells cocultured with TAMs-conditioned, exosome-depleted TAMs-conditioned media or TAMs-conditioned media pretreated with GW4869 under normoxia (F) and hypoxia (G). (H) Fold change of miR-223 in A2780 treated with RNA polymerase II inhibitor or PBS. (I-J) Related miR-223 level in macrophages (I) or exosomes derived from macrophages (J) treated with PBS, DMOG or YC-1 under normoxia and hypoxia. (K-L) A2780 cells were treated with exosomes derived from the normoxic or hypoxic macrophages which were transfected with agomir or antagomir respectively, the related miR-223 level was measured. (M) The number of cell colonies was detected in SKOV3 treated as indicated. *P < 0.05, **P < 0.01 (TIF 2315 kb) [file 13046_2019_1095_MOESM6_ESM.tif]

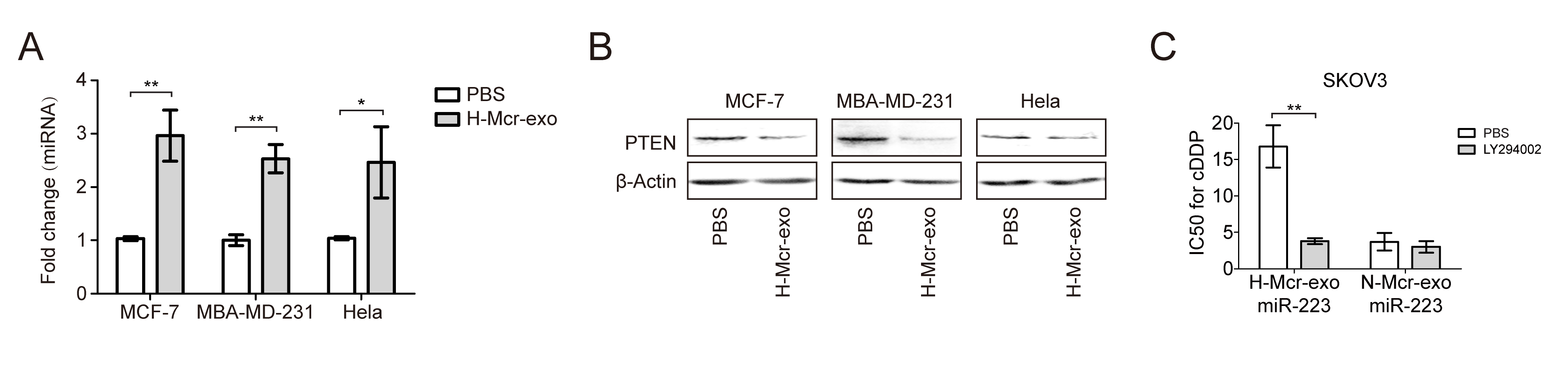

Supplement: Supplementary file 7 — Figure S5. (A-B) Related miR-223 (A) and PTEN protein (B) level in MCF-7, MBA-MD-231 and Hela cells treated with PBS or H-Mcr-exo, (C) IC50 for cDDP was measured in SKOV3 cells (pretreated with LY294002) cocultured with exosomes derived from the normoxic or hypoxic macrophages which were transfected with agomir. *P < 0.05, **P < 0.01 (TIF 395 kb) [file 13046_2019_1095_MOESM7_ESM.tif]

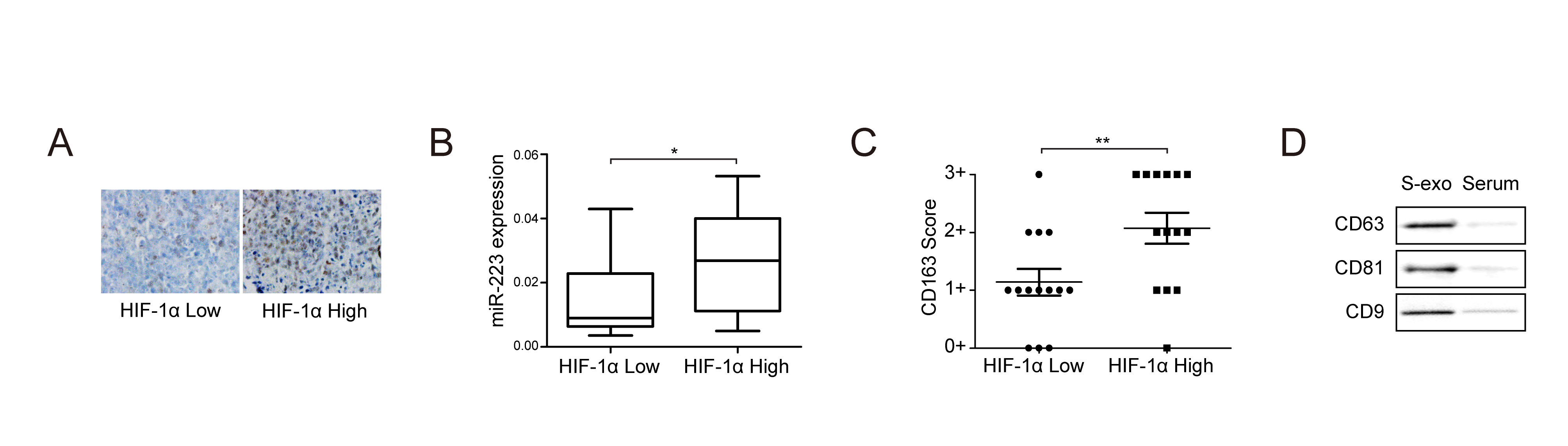

Supplement: Supplementary file 8 — Figure S6. (A) Representative images of low (left) or high (right) HIF-1a expression in EOC samples by immunohistochemical staining. (magnification, × 200). (B-C) Intertumoral level of miR-223 (B), and CD163+ cell infiltration (C) (representative of TAMs infiltration) were measured with high and low HIF-1a expression in 28 primary EOC tissues. (D) Representative images of CD81, CD63, and CD9 in serum and its derived exosomes from an EOC patient. *P < 0.05, **P < 0.01 (TIF 1007 kb) [file 13046_2019_1095_MOESM8_ESM.tif]
